# Quantitative characterisation of Quaternary glaciofluvial aquifer heterogeneity using cluster analysis

**DOI:** 10.1007/s10040-025-02933-z

**Published:** 2025-08-02

**Authors:** Felipe Gallardo Ceron, Landis Jared West, Ian T. Burke, James Graham, Luca Colombera

**Affiliations:** 1 Institute of Applied Geosciences, School of Earth and Environment, University of Leeds, Leeds, LS2 9JT UK; 2 Earth Surface Science Institute, School of Earth and Environment, University of Leeds, Leeds, LS2 9JT UK; 3 National Nuclear Laboratory Ltd., Sellafield, CA20 1PG UK; 4 Department of Earth and Environmental Sciences, University of Pavia, Pavia, Italy

**Keywords:** Hydraulic properties, Heterogeneity, Glaciofluvial, Clustering analysis, Kozeny–Carman

## Abstract

Hydrogeological modelling requires the characterisation of hydrofacies and the representation of their spatial distribution. In this work, a workflow was developed to characterise heterogeneous unconsolidated glaciofluvial sediments in Northwest Cumbria (UK), which involved: (1) field sampling; (2) lithofacies classification; (3) in situ porosity measurements using the sand-replacement method; (4) determination of the particle size distribution (PSD); (5) hydrofacies definition via K-means cluster analysis using PSD data; (6) evaluation of empirical equations for predicting porosity using field measurements and regression analysis; and (7) estimation of hydraulic conductivity (*K*) using the Kozeny–Carman equation. Nine lithofacies were identified, including glaciofluvial silts, sands and gravels, and local till deposits. Three clusters were defined on the basis of PSD: fine-dominated (cluster-1), sand-dominated (cluster-2) and gravel-dominated (cluster-3). Cluster-1 exhibited the highest porosities (average 44%); cluster-2 showed intermediate to high porosities, with an average porosity of 40%; and cluster-3 had the lowest porosities (average 27%). The logarithm of *d*_50_ was the parameter with the highest correlation with measured porosities (*R*^2^ of 0.789). *K* values estimated using the Kozeny–Carman equation ranged between 0.06 and 0.2 m/d for cluster-1, 0.2–11 m/d for cluster 2, and 0.1–62 m/d for cluster 3. Measured porosities were higher than previously reported, while estimated *K* values were consistent with those from hydraulic tests. Comparison between lithofacies and clustering classification suggests that, for hydrofacies classification, the unsupervised cluster analysis approach is able to generate a classification that captures the hydrogeologically important details without creating an excessive number of categories.

**Supplementary Information:**

The online version contains supplementary material available at 10.1007/s10040-025-02933-z.

## Résumé

La modélisation hydrogéologique nécessite la caractérisation des hydrofaciès et la représentation de leur répartition spatiale. Dans ce travail, une démarche a été développée qui vise à caractériser les sédiments fluvioglaciaires non consolidés et hétérogènes de la Cumbrie du Nord-Ouest (Royaume Uni), qui comprenait : (1) un échantillonnage de terrain;(2) la classification des lithofaciès; (3) la mesure in situ de la porosité par la méthode du remplacement de sable; (4) la détermination de la distribution de la taille des grains (DTG); (5) la définition des hydro-faciès à l’aide de l’analyse du cluster “conductivités hydrauliques moyennes” sur la base des données DTG; (6) l’évaluation des équations empiriques pour la prédiction de la porosité utilisant les mesures de terrain et les analyses de régression; et (7) l’estimation de la conductivité hydraulique (*K*) utilisant l’équation de Kozeny–Carman. Neuf hydrofaciès ont été identifiés, comprenant des silts fluvioglaciaires, des sables, des graviers et des dépôts locaux de till. Trois clusters ont été définis sur la base des DTG : à dominante de fines (cluster-1), à dominante de sables (cluster-2) et à dominante de graviers (cluster-3). Le cluster-1 a montré les porosités les plus fortes (moyenne de 44 %); le cluster-2 des porosités intermédiaires à fortes, avec une porosité moyenne de 40 %; et le cluster-3 les porosités les plus faibles (moyenne de 27 %). Le paramètre logarithme de *d*_50_présentait la corrélation la plus forte avec les porosités mesurées (*R*^2^ de 0.789). Les valeurs de*K* estimées d’après l’équation de Kozeny–Carman se répartissaient entre 0.06 et 0.2 m/j pour le cluster-1, entre 0.2 et 11 m/j pour le cluster-2, et entre1 et 62 m/j pour le cluster-3. Les porosités mesurées étaient plus fortes que celles rapportées précédemment, tandis que les valeurs de *K* estimées étaient cohérentes avec celles des tests hydrauliques. La comparaison entre les lithofaciès et la classification par cluster suggère que, en ce qui concerne la classification des hydrofaciès, l’approche par l’analyse non supervisée de clusters est capable de générer une classification qui saisit des détails hydrogéologiquement importants sans créer un nombre excessif de catégories.

## RESUMEN

El modelado hidrogeológico requiere la caracterización de las hidrofacies y la representación de su distribución espacial. En este trabajo se desarrolló un flujo de tareas para caracterizar sedimentos glaciofluviales heterogéneos no consolidados en el noroeste de Cumbria (Reino Unido), que implicó: (1) muestreo de campo; (2) clasificación de litofacies; (3) mediciones de porosidad in situ utilizando el método de reemplazo de arena; (4) determinación de la distribución granulométrica (PSD); (5) definición de hidrofacies mediante análisis de clúster *K*-medias utilizando datos PSD; (6) evaluación de ecuaciones empíricas para predecir la porosidad utilizando mediciones de campo y análisis de regresión; y (7) estimación de la conductividad hidráulica (*K*) utilizando la ecuación Kozeny–Carman. Se identificaron nueve litofacies, incluyendo limos glaciofluviales, arenas y gravas, y depósitos de till locales. Se definieron tres clústeres basados en la PSD: dominado por finos (cluster-1), dominado por arenas (cluster-2) y dominado por gravas (cluster-3). El cluster-1 mostró las porosidades más altas (media 44%); el cluster-2 mostró porosidades intermedias a altas, con una porosidad media del 40%; y el cluster-3 tuvo las porosidades más bajas (media 27%). El logaritmo de d50* d*_50_ fue el parámetro con mayor correlación con las porosidades medidas (*R*^2^ de 0,789). Los valores de *K* estimados mediante la ecuación de Kozeny–Carman oscilaron entre 0.06-0.2 m/d para el cluster-1, 0.2-11 m/d para el clúster 2, y 0.1-62 m/d para el clúster 3. Las porosidades medidas fueron superiores a las comunicadas anteriormente, mientras que los valores estimados de *K* fueron coherentes con los de las pruebas hidráulicas. La comparación entre la clasificación por litofacies y la clasificación por clústeres sugiere que, para la clasificación por hidrofacies, el enfoque de análisis de clústeres no supervisado es capaz de generar una clasificación que capta los detalles hidrogeológicamente importantes sin crear un número excesivo de categorías.

## Abstract (zh)

水文地质建模需要表征水力相并刻画其空间分布特征。本研究开发了一套针对英国西北部坎布里亚地区非固结冰水沉积物的非均质性表征流程，包括：（1）野外采样；（2）岩相分类；（3）采用灌砂法进行原位孔隙度测量；（4）粒度分布(PSD)测定；（5）基于PSD数据的*K*均值聚类分析定义水力相；（6）通过实测数据与回归分析评估孔隙度预测经验公式；（7）应用Kozeny–Carman方程估算渗透系数（*K*）。研究识别出9种岩相类型，涵盖冰水沉积粉砂、砂、砾石及局部冰碛物。根据PSD划分出三个聚类：细粒主导型（Cluster-1）、砂质主导型（Cluster-2）和砾石主导型（Cluster-3）。Cluster-1孔隙度最高（平均44%）；Cluster-2孔隙度为中高值（平均40%）；Cluster-3孔隙度最低（平均27%）。*d*_50_的对数值与实测孔隙度相关性最高（*R*^2^ =0.789）。Kozeny–Carman方程估算的*K*值范围分别为：Cluster-1 0.06-0.2 m/d，Cluster-2 0.2-11 m/d，Cluster-3 0.1-62 m/d。实测孔隙度高于文献值，而估算*K*值与水力试验结果吻合。岩相与聚类分类对比表明，就水力相划分而言，无监督聚类分析方法既能捕捉水文地质关键特征，又可避免产生过多分类。

## RESUMO

A modelagem hidrogeológica requer a caracterização de hidrofácies e a representação de sua distribuição espacial. Neste trabalho, foi desenvolvido um fluxo de trabalho para caracterizar sedimentos glaciofluviais heterogêneos não consolidados no noroeste da Cúmbria (Reino Unido), que envolveu: (1) amostragem de campo; (2) classificação de litofácies; (3) medições de porosidade in situ utilizando o método de substituição de areia; (4) determinação da distribuição granulométrica (DDG); (5) definição de hidrofácies via análise de agrupamento K-médias utilizando dados de DDG; (6) avaliação de equações empíricas para previsão de porosidade utilizando medições de campo e análise de regressão; e (7) estimativa da condutividade hidráulica (*K*) utilizando a equação de Kozeny–Carman. Nove litofácies foram identificadas, incluindo siltes, areias e cascalhos glaciofluviais e depósitos locais de tiltos. Três agrupamentos foram definidos com base na DDG: dominado por finos (Agrupamento-1), dominado por areia (Agrupamento-2) e dominado por cascalho (Agrupamento-3). O Agrupamento-1 exibiu as maiores porosidades (média de 44%); o Agrupamento-2 mostrou porosidades intermediárias a altas, com uma porosidade média de 40%; e o Agrupamento-3 teve as menores porosidades (média de 27%). O logaritmo de *d*_*50*_ foi o parâmetro com a maior correlação com as porosidades medidas (*R*^*2*^ de 0.789). Os valores de *K* estimados usando a equação de Kozeny–Carman variaram entre 0.06-0.2 m/d para o Agrupamento-1, 0.2-11 m/d para o Agrupamento-2 e 0.1-62 m/d para o Agrupamento-3. As porosidades medidas foram maiores do que as relatadas anteriormente, enquanto os valores de *K* estimados foram consistentes com aqueles dos testes hidráulicos. A comparação entre litofácies e classificação de agrupamento sugere que, para classificação de hidrofácies, a abordagem de análise de agrupamento não supervisionada é capaz de gerar uma classificação que captura detalhes hidrogeologicamente importantes sem criar um número excessivo de categorias.

## Introduction

Understanding and representing sedimentary and petrophysical heterogeneities of geological media remain vital tasks in subsurface studies. The effects of sedimentary heterogeneity are shown in many studies (e.g. Anderson 1989; Koltermann and Gorelick 1996; de Marsily et al. 2005; Lee et al. 2007; Ronayne et al. 2010; Savoy et al. 2017; Tahmasebi 2018), including those on transport modelling in groundwater systems (e.g. Zappa et al. 2006; dell’Arciprete et al. 2012; He et al. 2013; Bianchi and Zheng 2016; Montero et al. 2021), hydrocarbon reservoir characterisation (e.g. Sweet et al. 1996; El-Deek et al. 2017; Gray et al. 2022), and geothermal energy exploitation (e.g. Crooijmans et al. 2016; Liu et al. 2019).

In hydrogeological modelling, representing the spatial distribution of hydrogeological units and forecasting fluid flow and solute transport are activities that require good understanding of the geological media being modelled and its inherent heterogeneities (Koltermann and Gorelick 1996; Li et al. 2001; Savoy et al. 2017). Thus, it is paramount that simulations employ appropriate representations of the spatial distribution of hydrogeological units and their hydraulic properties, constrained by data obtained from relevant subsurface or analogue datasets. Model inputs need to be detailed enough to account for the heterogeneities of interest, but general enough to allow convergence to a solution of flow and transport equations. Particular challenges arise when heterogeneous media exhibit abrupt changes in petrophysical and hydraulic properties, when fractures and faults are present, and/or when processes related to the lithological composition of the aquifer occur, such as sorption and desorption, or reactions between the aquifer minerals and groundwater (Ronayne et al. 2010; Goltz and Huang 2017; Ewis et al. 2022). Failure to adequately represent heterogeneities can result in inaccurate estimations of flow and transport (Zappa et al. 2006; He et al. 2013; Kawo et al. 2023).

Simulations of the spatial distribution of hydrogeological units and their properties commonly use geostatistical—and more generally stochastic—modelling methods. These include object-based algorithms (e.g. Ronayne et al. 2010; Colombera et al. 2019), and methods based on multiple point geostatistics (MPS; e.g. Montero et al. 2021; Kawo et al. 2023) or transition probabilities (e.g. He et al. 2013; Bianchi and Zheng 2016), among others. Thanks to computational advances, it is becoming increasingly easier, faster and more efficient to run stochastic modelling algorithms. However, the required inputs to apply any of those methods need to be geologically consistent and robust to obtain meaningful results.

Given that the permeability heterogeneity of sedimentary aquifers depends on lithofacies distributions (Klingbeil et al. 1999; Zappa et al. 2006; Kawo et al. 2023), defining lithofacies types or operative facies types and then grouping them into hydrofacies (hydrogeologically relevant lithological units) based on their estimated hydraulic conductivity is the first step in the construction of a groundwater model. To define and characterise those units, available geological data and hydraulic tests are regularly used.

Quaternary sedimentary successions, and particularly those containing glaciogenic deposits, represent a case in which sedimentological patterns are difficult to predict, given the high variability in lithology, geometry and topology of sedimentary units (McMillan et al. 2000; Lee 2018; Kurjanski et al. 2020; Smith et al. 2023). Where only borehole and outcrop data are available, other approaches are needed for site characterisation. Recent studies addressing this problem have followed a sedimentological approach founded on detailed facies and architectural-element analysis of outcropping deposits (Smith et al. 2023). This approach results in a conceptual model of aquifer heterogeneity that accounts for the processes involved in the formation of the deposits. However, an excessive level of detail is not ideal for hydrogeological modelling purposes (e.g. Hill 2006).

Modelling a heterogeneous sedimentary aquifer involves representing (1) the spatial distribution of hydrogeological units and (2) their hydraulic properties. To undertake these tasks, one option is to inform the categorization of the units and their petrophysical characterisation using the particle size distribution (PSD) of sedimentary deposits (Alyamani and Şen 1993; Arya et al. 1999; Odong 2008; Rosas et al. 2014; Bianchi and Zheng 2016; Chandel and Shankar 2022). In applications to classification problems, PSD data are especially useful for grouping samples collected for hydrogeological characterisation (Simon et al. 2021; Nichols et al. 2023), given the role of sedimentological properties as controls on hydraulic characteristics (e.g. Anderson et al. 1999). Hydraulic conductivity estimates using the PSD have been derived from empirical equations since the late 19th century (Hazen 1892; Kozeny 1927; Fair et al. 1933; Carman 1937, 1956; Vuković and Soro 1992; Kasenow 2002; MacDonald et al. 2012; Rosas et al. 2014; Wang et al. 2017; Sun et al. 2024).

The relationships between the grain-size distribution and the porosity have been studied by several authors (e.g. Vuković and Soro 1992; Wu and Wang 2006; Wooster et al. 2008; Frings et al. 2011). However, empirical equations may not be readily applicable to sediments with different characteristics and/or from different settings to those for which the relationships were defined. To partly address this problem, the grain-size parameter that best predicts the porosity of the studied glaciofluvial sequences can be identified comparing measured porosities with grain size data. In turn, aiming to obtain a reasonable number of hydrofacies, a comparison between a lithofacies classification, based on a detailed sedimentological criteria, and a classification obtained from an unsupervised clustering approach using the grain-size distribution can be carried out. Unsupervised clustering has the potential to generate groups from large datasets based on input variables, such as the grain size, without needing to label data in advance (Jain et al. 1999), hence generating data-driven labels and reducing subjectivity in the classification.

**Fig. 1:**
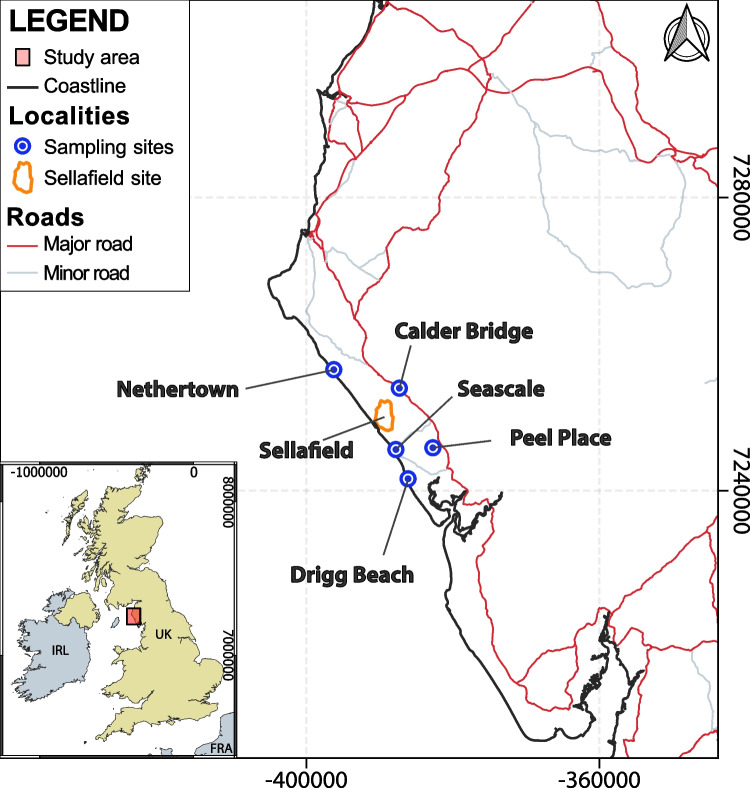
Map of the study area, NW Cumbria, UK. Sampled localities are shown. Grid geodetic reference provided in the World Geodetic System 1984 (WGS84) and Universal Transverse Mercator (UTM) coordinate system. Countries labeled following ISO-alpha3 M49 codes

In that context, the aim of this work is to develop a workflow for objective hydrofacies definition, applied to a heterogeneous unconsolidated glaciofluvial sequence in Northwest Cumbria (Fig. 1). Specific objectives include:

(i) Field characterisation of lithofacies in coastal and quarry outcrops, including field porosity measurements and sampling for laboratory analysis of PSD.
(ii) Identification of an optimum approach to estimate porosity from grain-size parameters where porosity is not measured in the field, as is generally the case given the challenges in undertaking porosity measurements.
(iii) Application of a clustering algorithm to PSD data to identify the optimum number of hydrofacies, and comparison with field observation-based lithofacies classification.
(iv) Definition of statistical hydraulic properties of the defined hydrofacies using estimates of the hydraulic conductivity based on PSD and porosity, and comparison to other data sources.

### Geological setting

The Quaternary geological history of northwest Cumbria (UK) was characterised by repeated advances and retreats of ice sheets during the Late Devensian (28–13 ka; Merritt and Auton 2000; Smith et al. 2020): main Late Devensian glaciation, Gosforth Oscillation, Main Scottish Readvance and minor readvances periods (Merritt and Auton 2000). This led to the deposition of heterogeneous, glaciofluvial sequences that constitute today's superficial, unconsolidated aquifer in the area. These deposits overlie Permo-Triassic sandstones of the Sherwood Sandstone Group, Ordovician volcaniclastic rocks of the Borrowdale Volcanic Group near the foothills to the east, and, locally, older Paleozoic rocks (Akhurst et al. 1997; McMillan et al. 2000; Merritt and Auton 2000).

Several studies have focused on the geological characterisation of the exposed Quaternary sequences (Akhurst et al. 1997; McMillan et al. 2000, 2011; Merritt and Auton 2000; Coleman et al. 2021; Smith et al. 2023), the past movement of ice sheets (Evans et al. 2005; Livingstone et al. 2012; Chiverrell et al. 2018), and the development of geological and hydrogeological models (McMillan et al. 2000; Smith et al. 2020, 2023; Coleman et al. 2021). Outwash gravel and sand deposits, of varying thickness, occur along the coast from Nethertown to Drigg Beach (Fig. 1). In the latter area, gravels are a subordinate component, and sands are dominant. Glacial till layers have also been recognised along this coast. Smith et al. (2023) characterised 19 different lithofacies that occur in five different facies associations (FA). The facies associations include sheet-like massive diamicts, gravelly confined flow and unconfined flow deposits, confined flow and lacustrine deposits, sand-dominated unconfined flow deposits and high-energy channelised flow deposits. The FAs map onto 11 types of architectural elements (AE). Glaciotectonic deformation has also been documented in these deposits. Overall, the deposits are heterogeneous in terms of their grain-size and spatial distributions, and their lateral extent is typically limited; as such, traditional approaches of borehole correlation for subsurface characterisation are problematic (McMillan et al. 2000; Smith et al. 2023).

Understanding sedimentological controls on contaminant migration in this aquifer is desirable, in the light of past leaks and spills of radionuclides into the subsurface of the Sellafield Nuclear Licenced site (Sellafield Ltd. 2023), and to support the safety case of the nearby UK Low Level Waste Repository (LLWR; LLW Repository Ltd. 2011). Sedimentary heterogeneities in this area determine the occurrence of preferential flow, especially via high-permeability gravel layers, but also spatial variability in sorption processes of contaminants such as ^90^Sr, which interacts with clays and iron oxides (Barker et al. 2024).

## Materials and methods

**Fig. 2:**
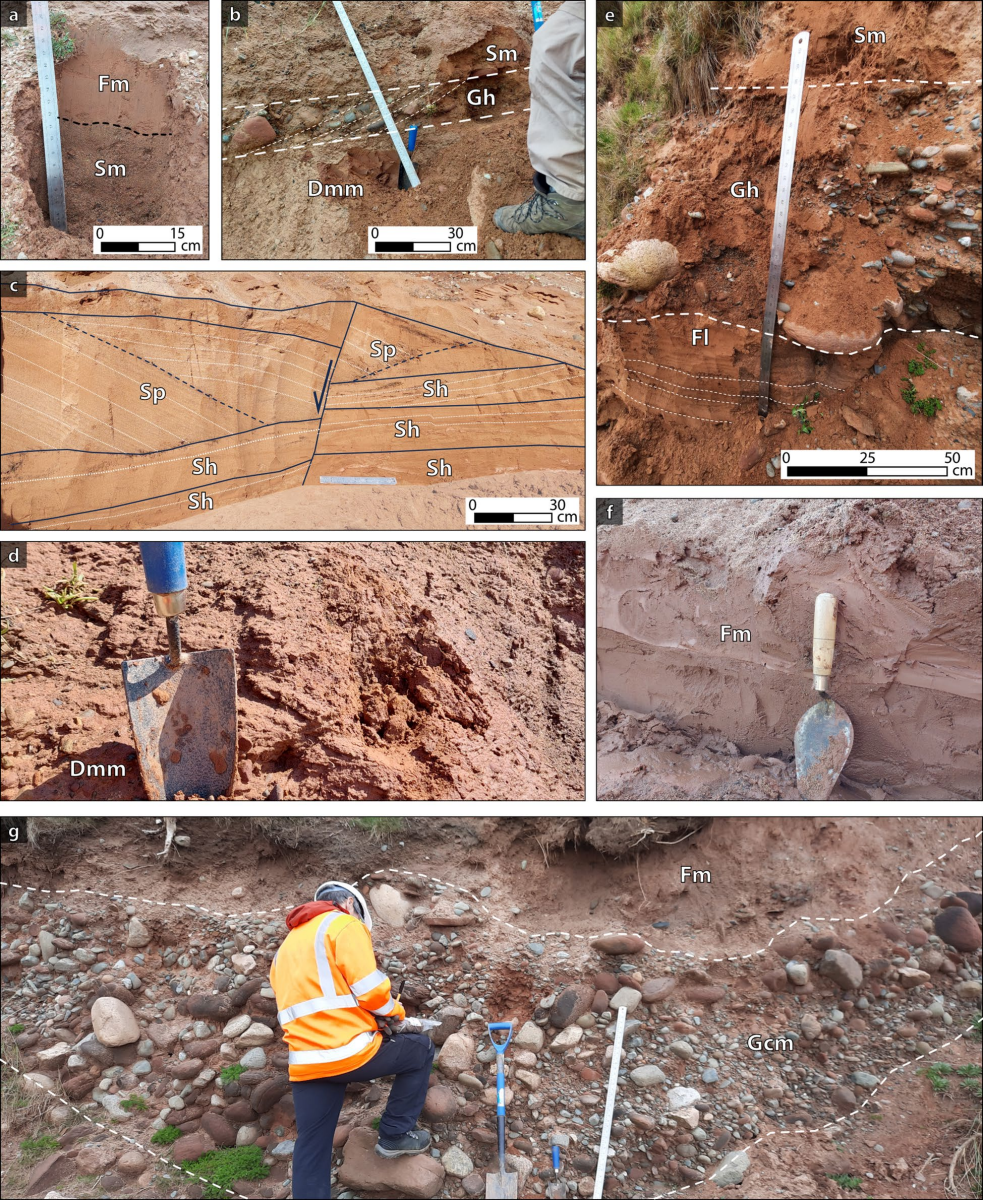
Outcrop photographs of facies characterised on the field. (**a**) Drigg beach, silt layer (Fm) overlying a massive sand (Sm). (**b**) Drigg beach, diamicton (Dmm) underlying a gravel layer with imbricated clasts (Gh), and a massive sand on top (Sm). (**c**) Peel Place quarry, horizontal and planar cross bedded sands (Sh and Sp), and a normal fault interrupting the continuity of the deposits. (**d**) Diamicton (Dmm). (**e**) Seascale, layered silt (Fl) overlain by an ~80 cm, fining-upwards, horizontally bedded gravel (Gh). A massive sand overlies the whole sequence. (**f**) Peel Place quarry, clayey silt (Fm). (**g**) Seascale, massive clast supported gravel (Gcm), folded, overlain by a massive silt (Fm). Photograph accreditation: Felipe Gallardo, Jared West

The identification and characterisation of lithofacies were undertaken through the following activities (the general workflow is shown in Fig. S1 of the electronic supplementary material, ESM): (1) field sampling at localities in the study area where fluvioglacial sequences are known to be exposed (Fig. 1); (2) lithofacies classification based on the visual characterisation of the deposits in the field; (3) in situ porosity measurements using the sand replacement method following British Standard BS1377-9 (British Standards Institution 1990); (4) determination of the particle size distribution (PSD) of the samples by combining mechanical sieving (> 2 mm fraction) and an optical grain sizer equipment (< 2 mm fraction); (5) data processing and data analysis, including an evaluation of empirical equations for predicting porosity (e.g. Vuković and Soro 1992; Wu and Wang 2006; Wooster et al. 2008; Frings et al. 2011) and determination of hydrofacies units via cluster analysis using the sample PSD data; and (6) hydraulic conductivity estimation using Kozeny–Carman equation (Kozeny 1927; Carman 1937, 1956; Bear 1972; Riva et al. 2010). A detailed explanation of each step is described in the following subsections.

### Fieldwork

Field sampling was carried out at the coastal outcrops of Drigg Beach, Seascale and Nethertown, and at Peel Place Quarry and the bank of the River Calder upstream of Calder Bridge (Fig. 1) following BS17892-4 (British Standards Institution 2016). Coastal outcrop sampling involved the systematic collection of sediment samples in vertical transects: four cliff sections were sampled at Drigg Beach and Seascale, and three at Nethertown (Table 1). Sampling intervals were identified on the basis of visual changes in the sedimentary composition of the sequence. The extent of vertical sampling was limited by outcrop accessibility. The steep nature of the outcrops precluded porosity measurements at these coastal locations. At Peel Place Quarry site, the stable nature of the slopes allowed sampling along both horizontal and vertical directions, and in situ porosity measurements using the sand replacement method. Three additional samples were obtained from outcrops of the glaciofluvial sequence along the Calder River, upstream of Calder Bridge. Pre-existing grain-size analyses from samples from two boreholes from the Sellafield site (9983 and 11 A), and Calder Bridge area, and of six further samples taken from Drigg Beach, Nethertown and Seascale Beach in March 2021 were included in the analysed dataset.

**Table 1:** Locations of samples described and obtained from the field (Field), and total number of PSD analyses (Lab). Grid references are provided in the Ordnance Survey National Grid reference system (OSGB)

Location	Grid reference (OSGB)	Number of samples	
		Field	Lab
Borehole 11A	NY 02304 04390^a^	1	4
Borehole 9983	NY 02304 04390^a^	2	8
Calder Bridge	NY 04221 06052	4	9
Drigg Beach	SD 04646 98646	37	88
Nethertown	NX 98733 07488	12	47
Peel Place	NY 06576 01310	18	51
Seascale	NY 03981 00402	15	38
Total	-	89	245

^a^Does not correspond to the exact borehole location but to a reference location of Sellafield site

Porosity measurements were performed by means of the sand replacement method. The process followed BS 1377–9 (British Standards Institution 1990) with calibration sand characteristics 100% passage through a 600 μm sieve and 100% retention on the 63 μm, large cylinder (200 mm diameter). The calibration sand density was estimated in the laboratory before and after the field study, using a ± 0.05 g precision scale, from which an average value of 1.49 g/cm^3^ was obtained (difference ± 0.01 between min and max values). A scale with a ± 100 g precision was used in the field to carry out the in situ weight measurements; the resulting error was < 1% porosity. A larger source of error arose from overestimation of the volume of the dug hole, resulting from loss of calibration sand between the cover plate and the surface around the hole (due to irregularities in the surface). The average gap was estimated as 10 mm, and error bars of ± 2% porosity were estimated by assuming minimum and maximum gaps of 5 and 15 mm, respectively. Samples were collected at each porosity measurement location to characterise moisture content and PSD.

### Facies analysis

A sedimentological lithofacies classification was carried out on the basis of field observations and photos taken from the sampling points, following the classification schemes by Tucker (2003) and Miall (2006) (Table 2). The facies codes and sedimentological terminology used are consistent with previous work on the same successions (e.g. Smith et al. 2023). The different types of lithofacies were interpreted in terms of formative depositional processes, and their assemblages were classified into facies associations according to their spatial association and architectural features exhibited in outcrop. The facies associations are interpreted in terms of local depositional environments. Individual samples were linked to their lithofacies and facies association, enabling correlation of sedimentological characteristics and hydraulic properties.

**Table 2:** Lithofacies codes for siliciclastic sediments (based on Miall 2006 and Tucker 2003)

Parameter	Code
Lithology	G: gravel S: sand F: fines D: diamicton
Depositional structure	m: massive p: planar cross-bedded t: trough cross-bedded r: ripple cross-laminated h: horizontal bedding l: laminated
Fabric	c: clast supported m: matrix supported

### Particle size distribution (PSD) analyses

To obtain the PSD (see detailed workflow in Fig. S2 of the ESM), sieving tests were carried out in accordance with BS 1377–2 (British Standards Institution 2022a). Samples were first oven-dried at 105 °C for 48 h. Coarse-grained samples (coarse gravels or total sample weight > 5 kg) were manually quartered before drying. After drying, the coarse fraction (> 2 mm) of each sample was separated from the fine fraction (< 2 mm) by mechanical sieving, and PSD analyses were performed separately on each fraction. The remaining fine fraction (< 2 mm) was then quartered using a riffle splitter. At least two separate fine-grained subsamples were obtained for each field sample. Given the limited size of the manual riffle, when the weight of the under 2 mm fraction was > 500 g, more pairs of subsamples were obtained. When the finer fraction was evidently poorly sorted and its weight was higher than ~500 g, an additional mechanical sieving step was carried out to obtain the < 1 mm portion. The resultant fine-fraction subsamples were analysed in the CAMSIZER® X2 optical sizer (Technology review 2013; Arora et al. 2022; Microtrac MRB 2024), which is based on the dynamic image analysis principle as presented in BS13322-2 (British Standards Institution 2022b). This equipment allows fast PSD analysis from a teaspoon-sized sample (between 20 and 50 g), for grain sizes < 2 mm. For most samples, the X-FALL module (dry dispersion by gravity - air; 10 μm to 3 mm) was used. For finest-grained samples, particularly those that agglomerated after drying, the X-JET module (dry dispersion by compressed air; 0.8 μm to 1.5 mm) was used as it deagglomerates the samples via a Venturi nozzle. Details on how each module work are explained in the manufacturer’s webpage (Technology review 2013; Microtrac MRB 2024). For each analysed sample, the CAMSIZER gives two PSD outputs: one considering the minimum diameter of each grain, and another one using the grain area. The former was used here as it is more comparable to mechanical sieving results.

### Data processing and analysis

Compiled PSD data was used to obtain the percentage of fine (< 0.0625 mm), sand (0.0625–2 mm) and gravel (> 2 mm) of each sample. These thresholds are widely recognised as key thresholds (Miall 2006; British Standards Institution 2018) and have been used for the sedimentological lithofacies classification of this work. Given that the same sample was analysed more than once, average values were obtained. The three grain-size fractions were plotted on a 2D ternary plot by reducing them to two independent variables using Eqs. [1] and [2],

1 $$X=\frac{1}{2}\bullet \frac{2\bullet \text{\%Gravel }+\text{ \%Fine}}{\text{\%Gravel }+\text{ \%Sand}+\text{ \%Fine}}$$

2 $$Y=\frac{\sqrt{3}}{2}\bullet \frac{\text{\%Fine}}{\text{\%Gravel }+\text{\%Sand }+\text{\%Fine}}$$

The resultant *X* and *Y* variables were used to classify the samples using the K-means algorithm. K-means is an unsupervised iteration-based clustering algorithm that stands out for its simplicity and ability to classify data points into clusters based on similarity (Jain et al. 1999; Pedregosa et al. 2011). It has been used, for example, to obtain lithofacies groups using seismic data (Troccoli et al. 2022) or geophysical logging (Newell et al. 2021) as input variables. It aims to minimise within-cluster distances and maximise between-cluster distances. It is initialised by setting the number of clusters then generating randomly positioned centroids to those clusters. Then, samples are assigned to their closest cluster (Euclidean distance), and centroids are recalculated. The algorithm iterates by reassigning samples their closest cluster considering the new centroids obtained. Finally, when no more samples are reassigned or centroids do not change anymore, the algorithm stops. To determine the optimum number of clusters, four methods were used. The elbow method plots the resultant sum squared error (SSE) against the number of clusters (the SSE is zero where the number of clusters equals the number of data points). The optimum number of clusters is that where the rate of reduction in SSE with increasing cluster number reduces (*elbow* in the plot). The silhouette method analyses the distance between clusters and number of samples contained in each (Rousseeuw 1987; Pedregosa et al. 2011). Plots are generated for different values of *n* (number of clusters) and a silhouette score is calculated. The optimum number of clusters is that when the average silhouette score is the highest, i.e. the clusters maximum silhouette coefficients are all above the average score, and cluster sizes are similar. The Davies–Bouldin index measures the similarity between clusters as the ratio between average within-cluster and between-cluster distances (Davies and Bouldin 1979; Pedregosa et al. 2011). A lower number means clusters are more separate from one another and less dispersed internally, which indicates the number of clusters selected is better. Finally, the Calinski–Harabasz criteria is defined as the ratio between the overall between-cluster variance and the overall within-cluster variance (Calinski and Harabasz 1974; Pedregosa et al. 2011). The result is higher when clusters have a lower within-cluster variance, and individual clusters are more separate between each other. The value will usually increase as the number of clusters increases, so algorithms usually look for the first local maximum to decide for an optimum number of clusters. Here, the Scikit-Learn Python package (Pedregosa et al. 2011) was used to run the K-means algorithm and obtain the elbow, silhouette, Davies–Bouldin and Calinski–Harabasz scores, with the optimum cluster number being found manually by comparison of efficacy of these four approaches.

Following the analysis of the ternary plot and the cluster analysis results, the grain-size variables required for subsequent porosity and hydraulic conductivity estimates were extracted and included in a database; these variables include the *d*_10_, *d*_25_, *d*_*5*0_ and *d*_75_ values; the uniformity coefficient ($U={d}_{60}/{d}_{10}$) and the geometric standard deviation (GSD) of *Φ*: $(\Phi ={-\text{log}}_{2}\left(\text{grain size}\right)$).

### Porosity and hydraulic conductivity estimation

Relationships were evaluated between porosity and *d*_50_ (following Wu and Wang 2006), the uniformity coefficient (*U*; following Vuković and Soro ( 1992)) and the geometric standard deviation of $\Phi$ (${\sigma }_{\Phi }$ or GSD*(Φ)*, following Frings et al. 2011; based on the equation by Wooster et al. 2008). A regression analysis was carried out and a site-specific calibration was found for each variable from which the approach showing the narrowest confidence intervals (least uncertainty) was selected. Porosity values were then estimated for the samples from outcrops where no direct porosity measurements were available, using that approach. These statistical porosity characteristics for each cluster were then defined using measured porosities (where available) and estimated values.

Hydraulic conductivity was then estimated from porosity and PSD data using empirical approaches. One of the most widely used is the Kozeny–Carman equation (K-C; Eq. 3), which relies on the porosity and on the representative grain size, and which has proven as a good fit for sediments similar to the ones being studied (Vuković and Soro 1992; Odong 2008; Chandel and Shankar 2022):

3 $$K=8.3\bullet {10}^{-3}\bullet \frac{\mathbf{g}}{\nu }\bullet \frac{{\theta }^{3}}{{\left(1-\theta \right)}^{2}}\bullet {d}_{\text{e}}^{2}$$

where $\mathbf{g}$ is the gravitational acceleration (9.81 m/s^2^), $\nu$ the viscosity coefficient of water (1.002 m^2^/s at 20 °C), $\theta$ the fractional porosity and *d*_e_ is the effective grain diameter (m). For this application, the characteristic grain size has been assumed to be *d*_10_ (following Vuković and Soro 1992; Kasenow 2002; Riva et al. 2010; Chandel and Shankar 2022).

## Results

### Samples and geological characterisation

A total of 81 samples were collected in the field and 12 different lithofacies have been identified (Table 3). A summary of the number of samples, locations and lithofacies is presented in Table 4.

**Table 3:** Sedimentological lithofacies characteristics

Facies code	Description	Interpretation (cf. Smith et al. 2023)
Diamicton Dmm (Fig. 2b, d)	Clayey-silt to gravelly silt diamicton. Polymictic, with subrounded to subangular clasts. Massive depositional structure. Brown colour. Very high stiffness	Subglacial till deposit (Evans et al. 2006; Smith et al. 2023)
Laminated silts Fl (Fig. 2e)	Sandy silt. Orange-brown, horizontally laminated. Bed thickness up to 0.2 m. Minor presence (< 1%) of subrounded clasts of up to 0.5 cm	Very low or inexistent flow, slack water deposit. Deposition from suspension (Bennett et al. 2002; Smith et al. 2023)
Massive silts Fm (Fig. 2a, f)	Silts and sandy silts. Occasional clasts (< 4%) up to 0.5 cm in size. Massive structure. Grey-brown to red-brown colour	Loess, overbank or abandoned channel fill deposits (Bennett et al. 2002; Miall 2006; Smith et al. 2023)
Massive sand Sm (Fig. 2a, b, e)	Very fine to coarse sands, well sorted. Massive, often normal graded. Occasionally with abrupt contacts between fine- and coarse-grained sands. Light brown to orange-brown colour. Occurs in units with thickness from 0.2 to 5 m	Deposition of sands out of suspension by energetic turbulent flows infilling channels (Smith et al. 2023)
Horizontally bedded sand Sh (Fig. 2c)	Fine to medium grained sand, well-sorted. Centimetric lamination. It is locally folded. It occurs in beds that have thickness from 0.5 to 2 m. Light brown to orange-brown colour	Unconfined flow events. Streamflow deposit under upper-flow regime conditions (Smith et al. 2023)
Planar cross-bedded sand Sp (Fig. 2c)	Very fine to medium sands. Planar cross-stratified. It shows intercalations of finer grained red-brown silty-sands. Mud drapes along the cross-stratified lamination. It may display centimetric scale folding. It occurs in units with thickness from 0.5 to 1 m. Orange-brown to light brown colour	Migration of straight-crested mesoforms, such as alluvial dunes, or linguoid and transverse bars (Miall 2006; Smith et al. 2023)
Massive clast-supported gravel Gcm	Sandy fine to coarse gravels. Poorly sorted, polymictic. Clasts size vary from 0.2 to 20 cm. Locally, clasts of up to 2 m size (Nethertown)	High energy mass flow and gravelly barforms (Miall 2006; Smith et al. 2023)
Planar cross-bedded gravel Gp	Sandy medium to coarse gravels with clasts that are up to 10 cm in diameter. Clast supported, poorly sorted, polymictic. Planar cross-bedded	Migration of gravelly barforms (Miall 2006; Smith et al. 2023)
Horizontally bedded gravel Gh (Fig. 2b, e)	Gravel and sandy gravels, with gravel-clast sizes varying mostly from 0.2 to 10 cm, but locally up to 50 cm. Poorly sorted, with polymictic subrounded clasts. Horizontally graded, locally imbricated	Fluvial sheet gravel or channel floor sediments in a fluvial environment (Miall 2006; Smith et al. 2023)

**Table 4:** Number of identified sedimentological facies per location

Location	Identified lithofacies									Total
	Dm	F		G			S			
	m	l	m	h	m	p	h	m	p	
Calder Bridge	-	-	1	-	2	-	-	-	-	3
Nethertown	1	-	2	-	4	-	1	-	1	9
Peel Place	-	-	1	-	6	-	4	4	3	18
Seascale	1	1	3	3	3	-	-	5	-	16
Drigg Beach	2	2	13	2	4	2	4	6	-	35
TOTAL	4	3	20	5	19	2	9	15	4	81

Sands and gravels are overall dominant (28 and 26 samples classified as such, respectively), while fines are slightly less abundant, having been recognised mostly at Drigg Beach (Table 4). Diamicton-type deposits (Dmm; Fig. 2b and d) have been observed at Drigg Beach, Seascale and Nethertown areas.

Gravel facies are pebbly, poorly sorted, clast supported and polymictic (Gcm, Gh; Fig. 2b, e and g). Clasts sizes are mostly in the 0.2 cm to 20 cm range, locally reaching > 1 m in diameter (e.g. Nethertown) and are dominantly red sandstone (presumably Sherwood Sandstone) and igneous (volcanic and occasionally granitic). Sand facies are mostly very fine to medium grained, well-sorted, occasionally containing sparse gravel-sized clasts (Sm, Sp, Sh; Fig. 2a and c). Fine-grained facies are represented by silts, clayey silts and sandy-silts, occasionally presenting clasts of up to 0.5 cm diameter (Fm, Fl; Fig. 2a, e, f and g).

Most of the studied deposits demonstrate a massive structure (Gcm, Sm, Fm; Fig. 2a and f), although horizontal stratification and cross-stratification are observed (Gh, Gp, Sh, Sp, Fl; Fig. 2b, c and e). Some gravel deposits show imbrication, with both a(t)b(i) (Fig. 2b) and a(p) fabrics (Fig. 2e).

Alternation of finer and coarser grained sized beds are observed at a scale of decimetres to metres (e.g. Fig. 2g); fining- and coarsening-upwards trends within beds are observed, although the former are dominant (Fig. 2). At Peel Place, local centimetric to metric scale faulting and folding are observed in sand-prone deposits (Fig. 2c). Larger-scale folds, of the order of metres to decametres, are evident in all coastal areas. Smith et al. (2023) attributed deformation features as such to be of glaciotectonic origin.

**Fig. 3:**
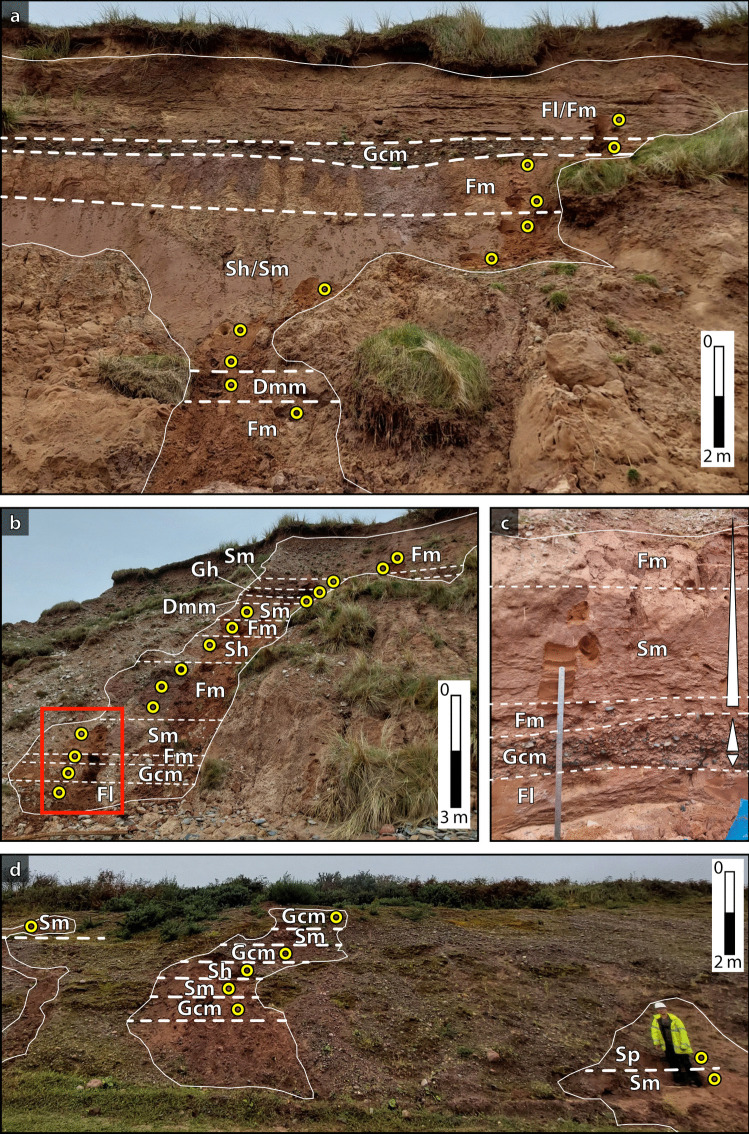
Facies associations observed in Drigg Beach (**a**, **b** and **c**) and Peel Place Quarry (**d**). Outcrops (**a**) and (**b**) are separated 100 m one from the other (south and north, respectively; Fig. 4). Highlighted rectangle in (**b**) is the area zoomed in (**c**). Sampling points are represented by yellow circles. Photograph accreditation: Felipe Gallardo

**Fig. 4:**
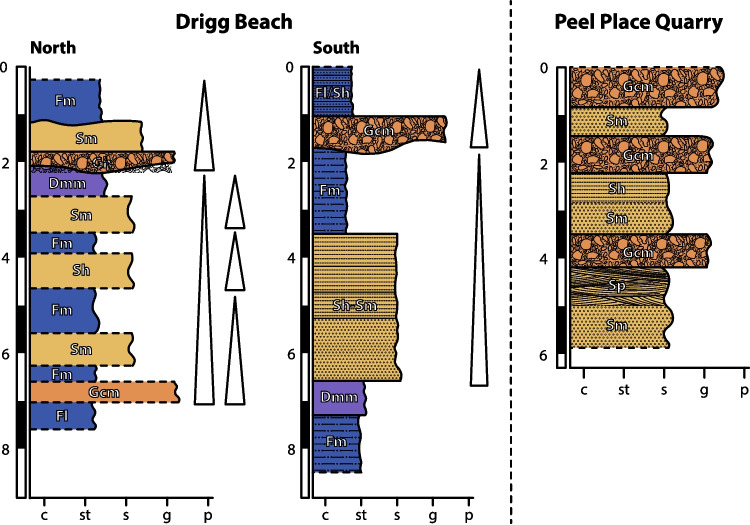
Schematic stratigraphic columns representing the deposit distribution in Drigg Beach and Peel Place Quarry

At Drigg Beach, the studied vertical transects conform to two main fining-upwards successions. Laterally, the succession varies in terms of the abundance and relative position of the different facies (Figs. 3a and b, and 4). The main facies types recognised correspond to massive clast-supported gravels (Gcm), horizontal plane-bedded and massive sands (Sh, Sm) and massive silts (Fm). A laminated sandy silt (Fl) is observed at the top and bottom of the exposed sequence, and an up to 1 m thick diamicton layer (Dmm) is observed along the sequence exposed in the south (Figs. 3a and 4). In the north exposure, the bottom fining-upwards succession is represented by an intercalation of silts and sands. In the base of the facies sequence, a laminated silt is overlain by a thin (< 50 cm) gravel bed (Figs. 3b and 4), which then shows a continuous, non-erosional, normal-graded sequence (Fig. 3c). The observations are consistent with the facies sequence being deposited in a glaciofluvial context, suggesting an origin related to stream-flow processes. The massive fine-grained deposits (Fm) with a predominance of silt and fine sand suggest the development of either loess, overbank or abandoned channel fill deposits. Smith et al. (2023) have characterised architectural elements in this area as sand-prone unconfined flow deposits (facies Sh, Sm), intercalated with unconfined flow gravels. Their fine-grained sequences in the area were attributed to subaqueous deposition in a glaciolacustrine environment, which could be consistent with the laminated silts and sandy silts observed. Diamictons observed by Smith et al. (2023) were interpreted as being deposited directly from glacier ice.

In Peel Place Quarry, the succession consists of coarse-grained, poorly channelized sand and gravelly deposits (Figs. 3d and 4). Facies recognised in this area include massive and horizontally laminated gravels (Gcm, Gh), and massive, horizontally bedded and planar cross-bedded sands (Sm, Sh, Sp). Smith et al. (2023) have characterised these facies as unconfined flow elements, interbedded with channel bar confined flow deposits, and solitary sand/silt fills. In general, they interpreted this facies sequence as gravel and sand dominated outwash deposits that might have accumulated under confined to unconfined flow conditions. At Seascale, the succession is dominated by gravels and sands, forming a facies association similar to that observed at Peel Place Quarry. However, massive and layered silts are also found along the facies sequence at Seascale. Layered silts are observed at the bottom of one of the vertical sections on this site; a massive silt overlies a clast-supported coarse gravel; and a massive fine-grained bed tops the succession. As there are no further exposures of the sediments on this site, it is unclear whether the overlying and underlying facies associations are similar to the one observed at Drigg Beach. Smith et al. (2023) interpret this sequence as being stratigraphically below the sequence observed in Peel Place Quarry.

### Particle size distribution

**Fig. 5:**
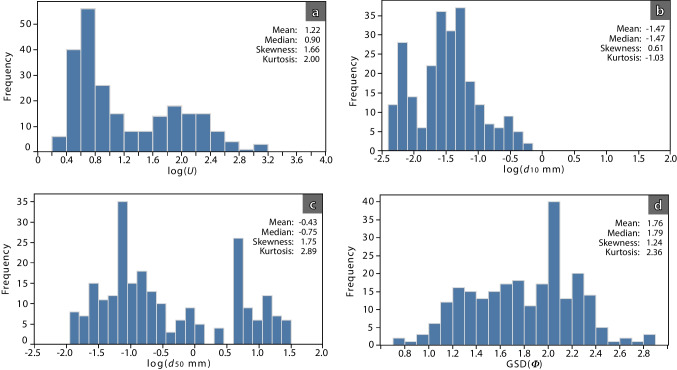
Frequency histograms: (**a**) log *U* (uniformity coefficient), (**b**) log *d*_10_, (**c**) log *d*_50_, (**d**) GSD(*Φ*), for the 245 analysed samples (from 89 sample locations)

The PSDs of 89 samples were obtained from a total of 245 analyses (Table 1). The frequency distributions of the summary statistics (*d*_10_, *d*_50_, GSD*(Φ)* and *U*) are shown in Fig. 5a to d, after log-transformation (base 10) for ease of visualisation. Distributions are all positively skewed; some grade of bimodality is observed in the *U*, *d*_10_ and *d*_50_ distributions, while GSD(*Φ*) shows a wider and more uniform dispersion of values. The uniformity coefficient (*U*) shows a high positive skewness with a median value of 0.90 for log *U* (Fig. 5a; *U* ~ 8), consistent with samples being predominantly poorly sorted. *D*_10_ is mostly distributed in the 0.01–0.1 mm range (log *d*_10_ between −2.0 and −1.0; Fig. 5b), showing a lower positive skewness. The mean and median values of log *d*_10_ are similar (−1.473 and −1.467, respectively) and the distribution can be approximated by a normal distribution, although a tendency to being bimodal is observed with a cluster of log *d*_10_ values between −2.5 and −2.0. In contrast, *d*_50_ shows a clear bimodal distribution (Fig. 5c), which also suggests that the uniformity coefficient distribution is more dependent on *d*_50_ than *d*_10_. The high skewness and kurtosis values are consistent with a wider distribution of this parameter. GSD*(Φ)* is also more evenly distributed (Fig. 5d), although its distribution is much more uniform, concentrated between 1.2 and 2.4. A major peak is observed at GSD*(Φ)* ~ 2.0.

## Analyses

### Cluster analysis

**Fig. 6:**
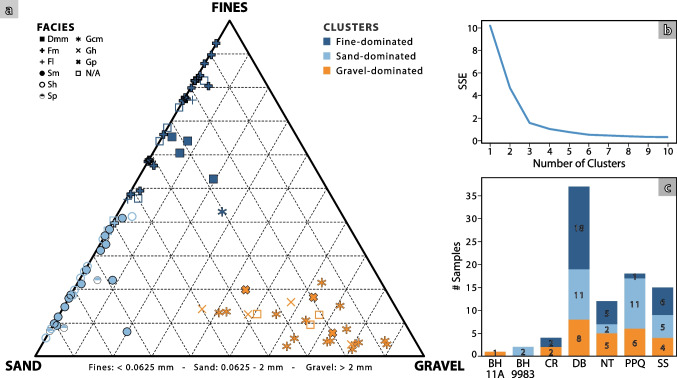
(**a**) Ternary plot showing the percentage of fines (< 0.0625 mm), sand (0.0625–2 mm) and gravel (> 2 mm) for each sampled location. Field lithofacies classification has been included as symbols, and colours represent the cluster in which samples have been classified. (**b**) Elbow method graph. (**c**) Number of samples per location (*BH* Borehole; *CR* Calder river; *DB* Drigg Beach; *NT* Nethertown; *PPQ* Peel Place Quarry; *SS* Seascale)

The resultant proportions of fines, sand and gravel are presented in the ternary plot of Fig. 6a. Samples with limited gravel fractions (< 10%) collectively cover the entire spectrum of sand- to fine-dominated deposits. Samples with a higher gravel content form a separate group, visually distinguishable in the plot, in which the sand content is < 60% and the content of fines is < 20%. Only a small number of samples are seen to plot in intermediate ranges between those two major groups.

**Fig. 7:**
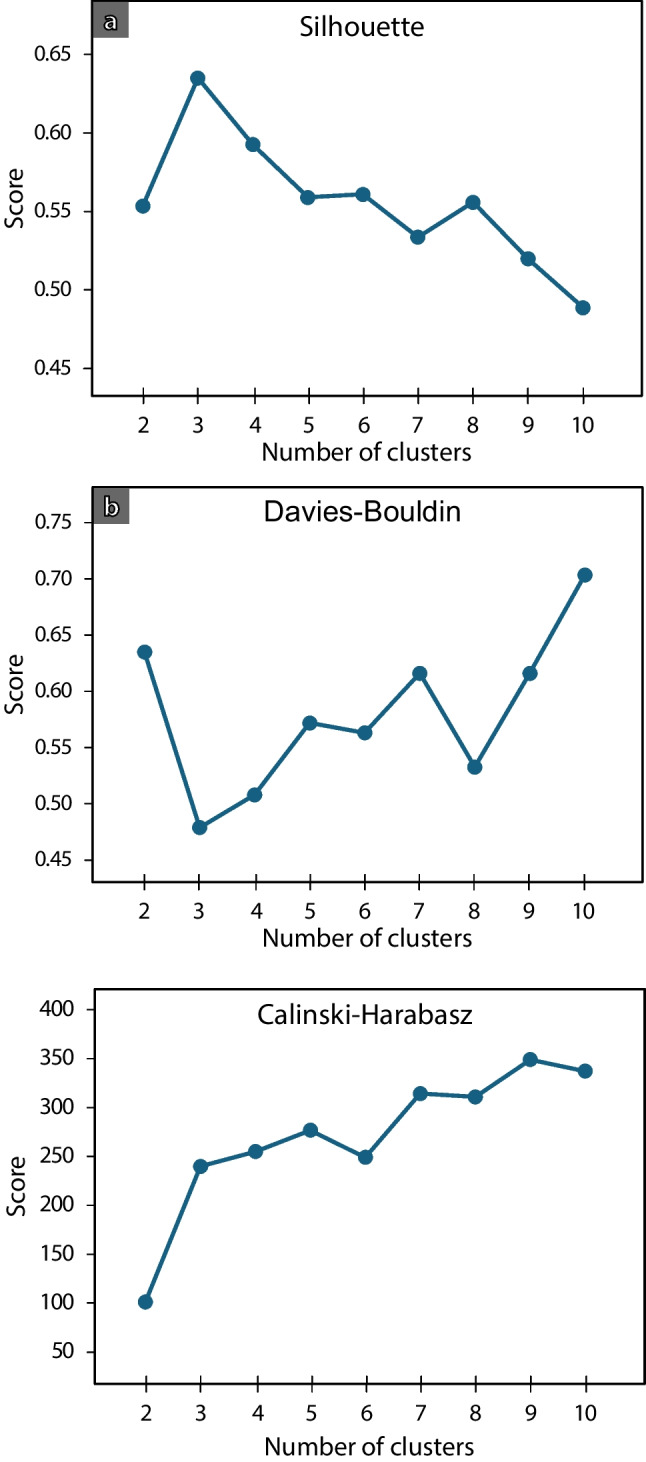
(**a**) Silhouette, (**b**) Davies–Bouldin and (**c**) Calinski–Harabasz scores for deciding the optimum number of clusters to use in the K-means algorithm

The four approaches described in the previous section were applied and their results manually compared to determine to optimum number of clusters. The elbow method suggested that three clusters are optimum (Fig. 6b). An optimum of three clusters is also supported by the silhouette and Davies–Bouldin maximum and minimum values, respectively (Fig. 7a and b). The Calinski–Harabasz score suggests five clusters as an optimum number, although three or four would also be acceptable. Given that three out of four methods strongly suggest three being the optimum number of clusters, three clusters were defined as follows: fine-dominated (32 samples; cluster 1), sand-dominated (31 samples; cluster 2) and gravel-dominated (26 samples; cluster 3) clusters (Fig. 6a). A summary of key statistics of each cluster and sample is included in the Tables S1 and S2 of the ESM).

**Fig. 8:**
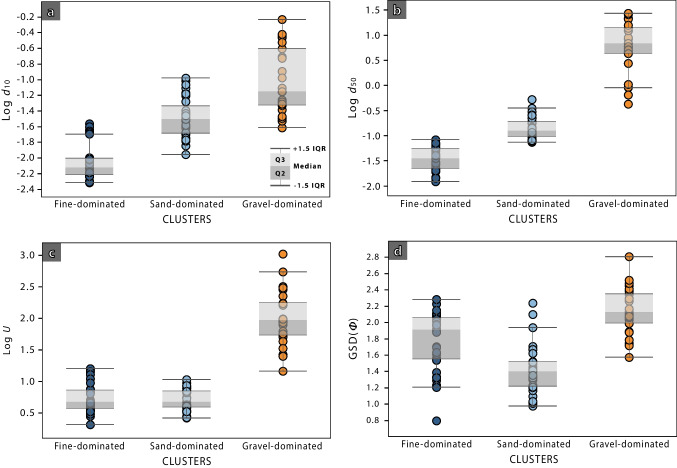
Distribution of (**a**) *d*_10_, (**b**) *d*_50_, (**c**) log *U* and (**d**) GSD(*Φ*) for each cluster

Fine- and sand-dominated clusters are characterised by a low content of gravel-sized grains: 3% and 2% on average, respectively. These clusters have the lowest *d*_10_ (Fig. 8a) and *d*_50_ values (Fig. 8b), with a group of values exceeding 1.5 times the interquartile range (IQR) observed in the fine-dominated (*d*_10_), and in the sand-dominated cluster (*d*_50_). They also have the lowest uniformity coefficients, with an average of 5.6 in cluster 2 and 6.3 in cluster 1 (Fig. 8c). The gravel-dominated cluster comprises the samples that were visually identified as a separate group (Fig. 6a). Fine-sized grains represent, on average, 10% of the samples belonging to the gravel-dominated cluster. In turn, the uniformity coefficient of this group is considerably higher than the other two, with an average of 175 and extreme values above 1000 (Fig. 8c). This is consistent with these samples being extremely poorly sorted. The GSD*(Φ)* shows a distribution more similar among the three clusters (Fig. 8d), although the sand-dominated cluster has lower values (average 1.4), and the gravel-dominated cluster has the highest ones (average 2.1).

### Porosity measurements and estimates

**Fig. 9:**
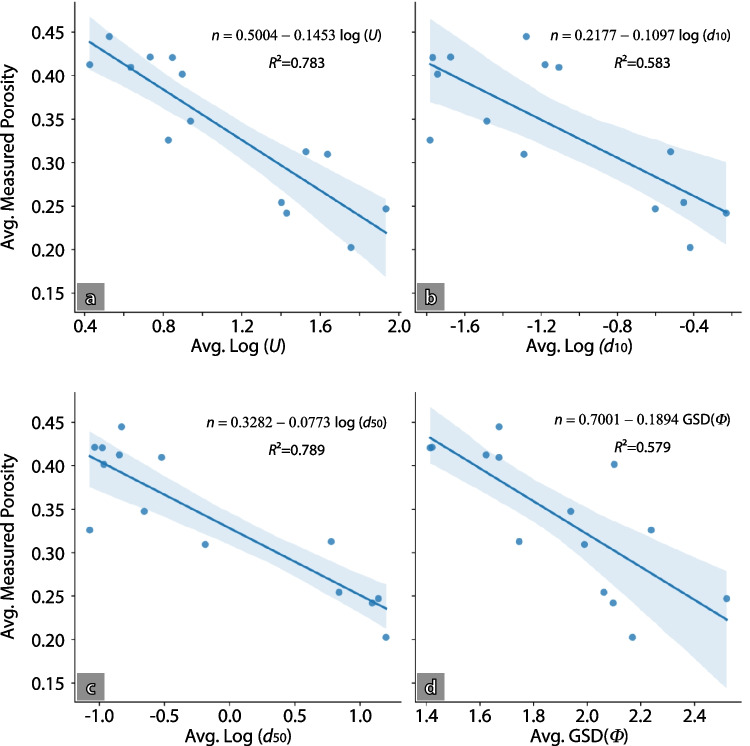
Scatter plots showing relationships between measured porosity (*n*) and particle size distribution parameters: (**a**) log *U*; (**b**) log *d*_10_; (**c**) log *d*_50_; (**d**) GSD(*Φ*). *Blue lines* indicate linear regressions (equation shown in each graph), whereas the *light blue fields* indicate the 95% confidence intervals

A total of 14 porosity measurements were obtained from deposits found at Peel Place Quarry site. These represent total porosities, which are equivalent to effective porosities when studying unconsolidated sediments (Woessner and Poeter 2020). Porosity from field measurements show a 35% average porosity, with a minimum value of 20% and a maximum value of 44.5%. Porosity measurements were plotted against the uniformity coefficient (*U*), the logarithm (base 10) of *d*_10_ (representative grain size used in hydraulic conductivity estimators); the logarithm of *d*_50_ and GSD*(Φ)*. The regression analysis shows that log *d*_50_ has the highest correlation with measured porosity values (Fig. 9), which is supported by log *d*_50_ having the highest Pearson’s correlation coefficient and coefficient of determination *R*^2^, and the lowest mean squared error and sum of squared errors (MSE and SSE; Table 5). In contrast, log *d*_10_ and GSD*(Φ)* show lower correlation with porosity. Overall, log *d*_50_ is identified as the variable that can be most confidently used to estimate the porosity of the remaining samples using equation [4]:

**Table 5:** Correlation coefficients between measured porosity and log *U*, log *d*_10_, log *d*_50_ and GSD(*Φ*). ${\sigma }_{\Phi }$: geometric standard deviation of *Φ*

Statistic	log *U*	log *d*_10_	log *d*_50_	GSD(*Φ*)
Pearsons	−0.885	−0.764	−0.888	−0.758
Spearmans	−0.859	−0.644	−0.798	−0.767
Best fit equation	$n=-0.1453\text{log}\left(U\right)+0.5004$	$n=-0.1097\text{log}\left({d}_{10}\right)+0.2177$	$n=-0.0773\text{log}\left({d}_{50}\right)+0.3282$	$n=0.975{\text{e}}^{-0.562 {\sigma }_{\Phi }}$
SSE	0.0183	0.0352	0.0178	0.0356
MSE	0.0015	0.0025	0.0014	0.0025
*R*^2^	0.783	0.583	0.789	0.579

4 $$n=-0.0773\bullet \text{log}\left({d}_{50}\right)+0.3282$$

**Fig. 10:**
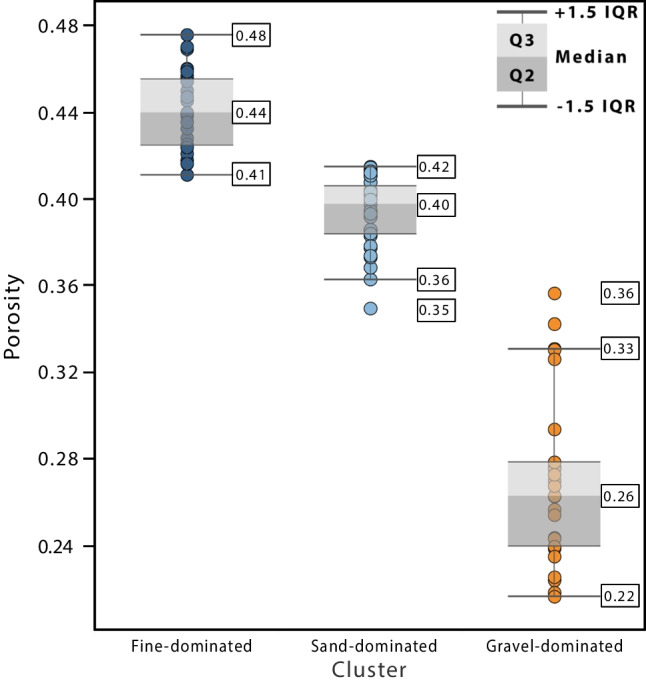
Distribution of porosity values per cluster using porosity *d*_50_ equation

Porosity estimates using this relationship (Fig. 10) show that the fine-dominated cluster has the highest porosity range (average 44%, range 41–48%), consistent with it corresponding to well-sorted fine-grained sediments. The range is slightly lower in the sand-dominated cluster (average 40%, range 35–42%), whereas the gravel-dominated cluster shows the lowest porosities (average 27%, range 22–36%). This is also consistent with the latter being poorly sorted (high *U*).

### Hydraulic conductivity estimates

**Fig. 11:**
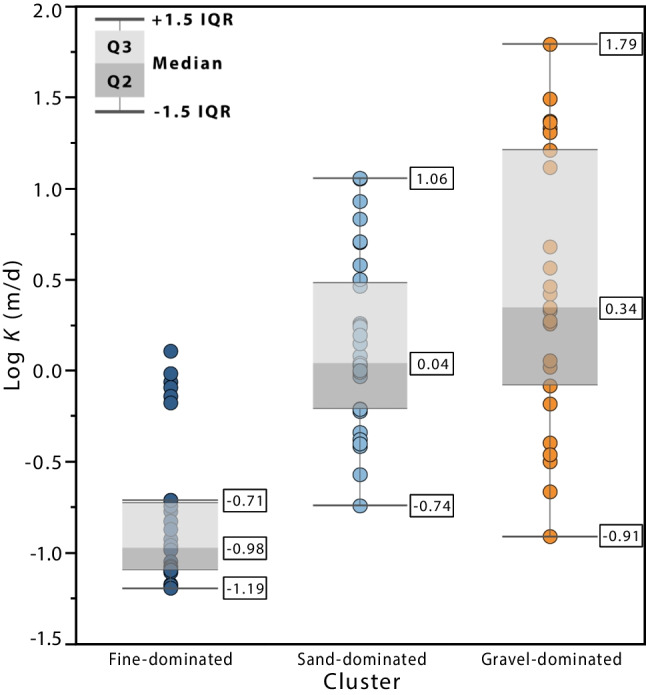
Distribution of hydraulic conductivity estimates, per cluster, using the Kozeny–Carman equation. Porosity estimates using the *d*_50_ equation were used as an input

The Kozeny–Carman equation was used to estimate the hydraulic conductivity (*K*) of the 89 samples. Figure 11 shows the distribution of log(*K*) for each cluster. The gravel-dominated cluster shows the highest median and geometric mean values of hydraulic conductivity (2.2 and 2.8 m/d, respectively) and the largest ranges of values (from 0.1 to 62 m/d). The sand-dominated cluster has a lower median and geometric mean than the gravel-dominated cluster (1.1 and 1.4 m/d, respectively), and a narrower range, from 0.2 to 11 m/d. The fine-dominated cluster shows a hydraulic conductivity range that is one order of magnitude lower, although six outlier samples have a *K* range overlapping with the second quartile of cluster 2 (sands). These outlier samples have relatively high *d*_10_ values (see outliers for fine-dominated cluster in Fig. 8a), which explains their higher *K* estimates. This is consistent with these samples having been analysed using the X-FALL module instead of the X-JET, as was done with all the other fine-grained samples. Hence, they should be discounted in any characterisation of clusters’ *K*-ranges. Treating these samples as outliers, *K* values for the fine-grained cluster are in the 10^–2^–10^–1^ m/d range (0.06–0.2 m/d; geometric mean of 0.1 m/d), while both sand- and gravel-dominated clusters have a range between 10^–1^ and 10^1^ m/d, with the latter being closer to the 10^2^ m/d limit. All the log(*K)* distributions show positive skewness.

## Discussion

### Cluster analysis and lithofacies

**Fig. 12:**
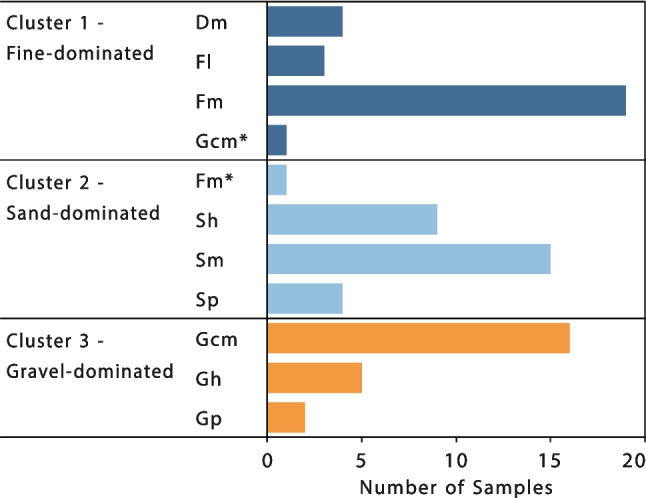
Frequency histogram of samples by lithofacies, per cluster. Gcm* and Fm* classified as cluster 1 and cluster 2, correspond to two samples located close to the boundaries of the corresponding clusters

**Fig. 13:**
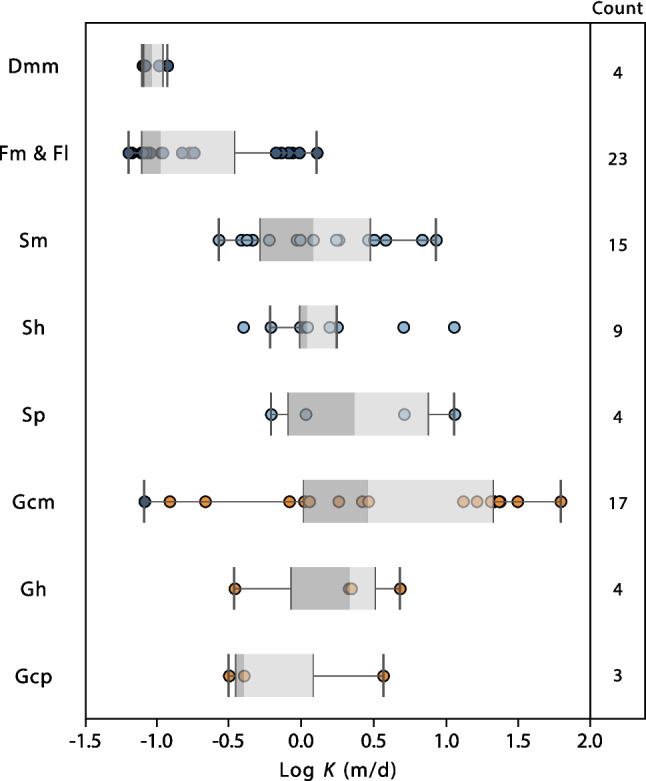
Distribution of log of hydraulic condictivity, estimated using the Kozeny–Carman equation, for each lithofacies

Clusters obtained from the PSD are consistent with field characterisation of lithofacies (Figs. 6 and 12). For instance, all the ‘G’ coded lithofacies fall into the gravel-dominated cluster, except for a single sample, identified as Gmm, which falls within cluster 1 (dark blue asterisk in the middle of the ternary plot; Fig. 6). Similarly, all of the ‘S’ coded lithofacies fall into cluster 2 (circles; Fig. 6) and all of ‘F’ coded and ‘D’ coded lithofacies fall into cluster 1 (dark blue crosses and squares; Fig. 6), with the exception of a single sample—coded F – light blue cross—which is close to the sand-fines midpoint on the ternary plot. This is an important finding because it highlights how the approach followed for lithofacies characterisation is based on objective observations made in the field, but that the statistical analysis based on laboratory PSD alone replicates the key lithotextural classes of fines/diamicton, sands and gravels without expert knowledge. Note that cluster analysis was unable to distinguish ‘D’ – diamicton, from ‘F’ – fines coded samples, as these plot relatively close together on the ternary plot. However, the d_10_ and porosity values for ‘F’ and ‘D’ samples are relatively similar, as are their predicted hydraulic conductivities (Fig. 13). Hence, while the samples are geologically distinct, it is appropriate to treat them as a single hydrofacies unit. In this sense, the unsupervised cluster analysis proves itself useful and efficient to classify lithofacies using the PSD and focusing on hydrogeologically important details, without creating an excessive number of categories.

Additional measurements, such as geochemical analyses (Simon et al. 2021; Nichols et al. 2023) or geophysical logging (Al-Mudhafar 2017; Kumar et al. 2022) could also be used for scopes of cluster analysis together with the PSD. Such data can help differentiate deposits. However, given the difficulty in obtaining these data from subsurface studies, using available grain size data in the manner explained in this work remains a more practical and broadly applicable approach to the characterisation of hydrogeological units.

The number of clusters used in the algorithm must align with the aim of the project rather than solely adhering to a specific criterion. For instance, the Calinski–Harabasz criterion (Fig. 7a; Calinski and Harabasz 1974) suggests an optimum of five clusters from the dataset, instead of three as suggested by the elbow method, silhouette analysis and Davies–Bouldin score. However, increasing the number of clusters reduces the correspondence with lithofacies, and does not provide additional hydrogeological or sedimentological distinction (the bottom-level lithofacies classes are not visually clustered, Fig. 6a). Notably, including log *d*_50_ as an additional third variable in the cluster analysis makes the number of clusters recommended on the basis of the Calinski–Harabasz criterion decrease to three, aligning with the number selected.

### Porosity and hydraulic conductivity estimates

To determine the most accurate porosity estimation equation, a pairwise bivariate analysis was followed using measured porosities and separate parameters (log *d*_10_, log *d*_50,_ log *U* and GSD*(Φ)*). The best-fit equation was obtained using d_50_ (Eq. 4). As expected, a multiple linear regression (MLR) results in an even better fit than a bivariate one, which is achieved using log *U*, log *d*_50_ and GSD*(Φ)* together (equation [5]; *R*^2^ = 0.899, MSE = 0.0006, SSE = 0.008):

5 $$n=0.53-0.04\bullet \left(\text{log}\left(U\right)+\text{log}{d}_{50}+2\bullet \text{GSD}\left(\Phi \right)\right)$$

However, the improvement in predictive power over the use of log *d*_50_ alone is marginal. When applying the MLR equation to the samples where porosity was not directly measured, some porosity estimates were significantly lower than any of the measured values. This happened in samples with high uniformity coefficient and *d*_50_ (exceeding 80 and 4 mm, respectively). Furthermore, for a few samples predicted porosities exceeded 50%, which is physically unrealistic for these sediment types. Given these considerations, the relationship between porosity and log *d*_50_ (Fig. 9c) has been used for estimating the porosity where it was not measured.

Porosity estimates obtained using empirical equations proposed in earlier studies (Vuković and Soro 1992; Wu and Wang 2006; Wooster et al. 2008; Frings et al. 2011) reflect known relationships between porosity and the considered PSD variables (*d*_10_, *d*_50_, GSD*(Φ)* or *U*). However, the application of those equations did not accurately match the measured values in this study. This highlights how empirical best-fit equations derived from other areas may not be universally applicable.

Using empirical equations to estimate the permeability of hydrogeological units is commonly done in practice when data are limited. McMillan et al. (2000) used the equation by Fair and Hatch (Fair et al. 1933) to estimate *K* values on glaciofluvial and glaciolacustrine sediments from the Sellafield area, whereas Bianchi and Zheng (2016) used the Kozeny–Carman equation to estimate the hydraulic conductivity of their lithofacies and for modelling a *K* field in glaciofluvial sediments from North America (MADE site, Mississippi, USA). As empirical approaches do not always result in a good match with measured values, it becomes important to know the applicability of different equations to different depositional environments (Rosas et al. 2014). In that sense, the Kozeny–Carman equation has proven to generate better estimates than other equations when used in sediments similar to the ones being studied (Odong 2008; Rosas et al. 2014; Chandel and Shankar 2022). To further support its usage, the applicability of the Kozeny–Carman equation was checked via comparison with hydraulic conductivities derived by other approaches and field data.

**Fig. 14:**
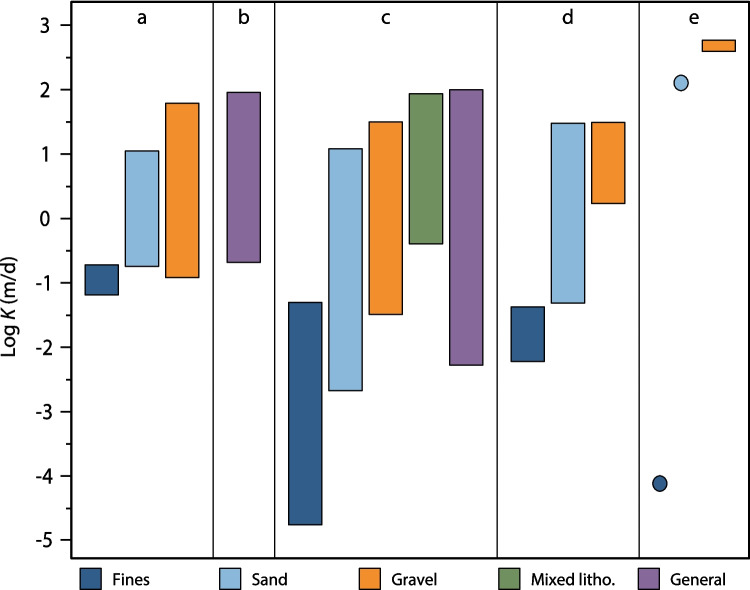
Ranges of values of log *K* (m/d) for different sediment sizes, according to different authors: (**a**) This work; (**b**) Nirex (1997b); (**c**) McMillan et al. (2011), including field measurements and PSD-based estimates as reported in Nirex (1997a); (**d**) MacDonald et al. (2012), including only measurements on glaciofluvial and glaciolacustrine deposits; (**e**) Purkis et al. (2023). Bars represent the whole range (min to max), while dots represent single measurements

Several studies have reported *K* values for the study site (Nirex 1997a; McMillan et al. 2000; Smith et al. 2023). The ranges of these *K* values are summarised in Fig. 14 and tabulated in Table S3 of the ESM. The *K* estimates for the sand-dominated and gravel-dominated clusters using the Kozeny–Carman equation show a good correlation with *K* measurements made by Nirex (1997a; reported in McMillan et al. 2000) on sand and gravel deposits using cone penetrometer, slug and pumping tests. The fine-dominated cluster has a geometric mean *K* value of 0.1 m/d, which is less than one order of magnitude higher than the estimated geometric mean of Nirex (1997a) obtained through a pumping test on a silt channel facies (0.019 m/d). Estimates reported in McMillan et al. (2000) using Fair and Hatch equation (Fair et al. 1933) are 0.5 to 3 orders of magnitude lower than the estimates presented in this work, which are considered unrepresentative.

Smith et al. (2023) reports a range of *K* values for each lithofacies (following LEwis et al. 2006 rock-types ranges). The values are useful for comparisons between different units; notably, it is observed that the predictions from this study are contained in the ranges reported. However, these ranges are for a very broad variety of geological materials and therefore do not provide useful site-specific information. Purkis et al. (2023) have recently measured the permeability of gravel and sand samples from the Peel Place Quarry site using the constant head method. Their results are one to two orders of magnitude higher than the estimates in this study. This is likely because their samples were re-compacted from loose sediment and hence had much higher porosities than in situ sediments, as well as no sedimentary structures. *K* ranges can also be determined during tasks of conceptual modelling and hydrogeological modelling calibration, when it is useful to have a reference range of values supported by appropriate analyses. Extensive hydrogeological flow modelling of the superficial aquifer from the Sellafield site produces overall hydraulic conductivities in the range 0.2 to 95 m/d (Nirex 1997b), with a geometric mean of 1.9 m/d. This range is comparable to the values predicted in this work using the PSD and Kozeny–Carman equation, although closer to the ones estimated for the sand-dominated and gravel-dominated clusters. This finding likely arises from the fact that modelled values represent the hydraulic conductivity of the sequence, which are dominated by the more permeable units (arithmetic weighted average).

Nevertheless, incorporation of the less permeable units represented by cluster 1 (fine-dominated) may be necessary to correctly model solute transport. Finally, MacDonald et al. (2012) measured the permeability of glacigenic deposits in Northern Scotland using a Guelph permeameter. Considering only the deposits they classified as glaciofluvial and glaciolacustrine, their estimated *K* ranges and median values for their sands (median ~2.5 m/d) and gravels (median ~5.5 m/d) are in the same order of magnitude as those obtained in this work (Fig. 14). Their silt deposits show a median *K* one order of magnitude lower (median 0.02 m/d) than this work. However, only two samples, one glaciolacustrine and one glacial till, have been included in this estimate, and their sand and silt classification might not exactly fit the one obtained through cluster analysis.

Using the Fair and Hatch equation (Fair et al. 1933; Rosas et al. 2014) instead of Kozeny–Carman, with the dataset here presented, leads to lower K estimates. Sand and gravel hydraulic conductivities are one to three orders of magnitude lower (Fair and Hatch ranges 2 × 10^–3^ to 3 × 10^–1^ m/d and 4 × 10^–4^ to 4 × 10^–1^ m/d, respectively). Estimates for the fine-dominated cluster are also lower than Kozeny–Carman estimates and better match other authors’ ranges, with values in the 10^–3^ to 10^–2^ m/d range. However, the method relies on the sum of the ratio between the percentage of grains retained between adjacent sieves and the geometric mean of the sieve’s sizes, and changing the number of sieves used can affect the hydraulic conductivity by up to one order of magnitude.

### Implications for hydrogeological modelling in heterogeneous porous media

Hydrogeological modelling requires definition of hydrofacies on a scale appropriate for the problem. For all but the smallest site-scale problems, a lithofacies classification that is too detailed might not be optimal for definition of hydrofacies, and some generalisation might be preferable whereby lithofacies units that have similar hydrogeological characteristics are grouped together into a single hydrofacies. A methodology that uses PSD data only is presented here to define hydrofacies using an unsupervised cluster-analysis approach. This methodology was tested against conventional classification based entirely on field outcrop observations. The agreement between cluster analysis and top-level lithofacies characteristics (F, S, G, D) was necessarily only partial, since the cluster analysis grouped D (diamict) together with F (fines). However, given that the predicted hydraulic characteristics of the latter two categories are similar, it is entirely appropriate to group them as a single hydrofacies for the purpose of hydrogeological modelling. Using the PSD data thus allowed solving two problems simultaneously: defining hydrofacies units and characterising their hydraulic properties. Such an approach has the advantage that it could be based entirely on borehole sampling, where, as is usually the case, no outcrop evidence is available. In this case study, no significant grouping pattern was observed between hydrogeological parameters and bottom level lithofacies indicators groups (m, c, p; i.e. those based on sedimentary structure within the top-level—particle size based—groupings F, S, G, D). However, it is possible that this may be an effect of the approach used to predict hydraulic conductivity, which used only *d*_10_ and porosity. Where there are additional variations in sediment texture and structure that may influence the hydraulic conductivity, additional variables needed to capture such features should be included in the cluster analysis. Using PSD data remains, however, a critical step, in view of the strong relationships between this and other petrophysical properties, sedimentological facies and depositional context.

The results presented in this work can be used as in dell’Arciprete et al. (2012) and Bianchi and Zheng (2016). They conducted a lithofacies characterisation of a heterogeneous porous media. Then, they used it as an input to generate realisations of the spatial distribution of the lithofacies and their hydraulic conductivity using the Markov chain/transition probability method (MC/TP; TPROGS code from Carle 1999). That same approach is suitable to the Sellafield site given the heterogeneous nature of the sediments at this site. Following this approach, several realisations can be generated to represent the spatial distribution of the lithofacies while keeping their connectivity as well as their heterogeneous distribution. PSD data from Sellafield site boreholes can be used to assign samples to the clusters identified in this work, which represent the appropriate hydrofacies to be used as an input for such geostatistical methods. Additionally, given that *K* ranges obtained for cluster 2 (sand-dominated) are contained within the range of those for cluster 3 (gravel-dominated), a potential bimodal hydrofacies definition could be tested on a hydrogeological model, as has been done by Bianchi and Zheng (2016).

Analogously, the workflow presented in this study is widely applicable to other sites in which sedimentary heterogeneities impact groundwater flow and contaminant transport, where inputs for hydrogeological modelling are needed, and where particle size distribution data are available. Heterogeneous fluvial sediments are widespread in terms of resource geology contexts, and simulations for hydrocarbon extraction, carbon capture and storage, hydrothermal potential, and general groundwater contaminant transport would potentially benefit from the approach described. Using the Kozeny–Carman equation to obtain hydraulic conductivity ideally requires either porosity data, or a site-specific evaluation of the relationship between PSD and porosity so that the latter variable can be inferred. Other empirical equations such as Hazen’s rule, the Terzaghi or Beyer equations, which do not need porosity, may be applied instead of Kozeny–Carman where this can be justified in terms of the grain-size characteristics of the sediment (Rosas et al. 2014).

## Conclusion

An unsupervised hydrofacies identification approach was evaluated on a heterogeneous unconsolidated glaciofluvial sequence. The approach used the particle size distribution (PSD) with K-means unsupervised clustering algorithm. Sedimentological facies were defined from field observations and compared against the identified hydrofacies from cluster classification. Three clusters—fines/diamicts, sands and gravels—were defined using the K-means algorithm. Results showed excellent correlation between sedimentological lithofacies and cluster analysis. Although each cluster typically contained multiple sedimentary facies, these had essentially similar predicted hydrogeological parameters. For instance, both fines-only and diamicton facies fell within the fine-dominated cluster, but these lithofacies had similar *d*_10_ grain size and, therefore, similar predicted hydraulic conductivities. For hydrogeological classification, this unsupervised cluster analysis approach is simpler to apply than lithofacies classification; it captures hydrogeologically important details without creating an excessive number of categories, does not require the same level of expert knowledge to apply and reduces observational bias while remaining consistent with a sedimentological lithofacies classification.

The workflow developed here used a limited number of field porosity measurements (using the sand replacement method) to correlate those porosities to various grain-size characteristics (*d*_10_, *d*_50_, *U*, geometric standard deviation of *Φ*). From these, log *d*_50_ showed the best correlation with the measured porosities. Porosities are higher than previously reported by Nirex (1997a) and used in subsequent works (e.g. McMillan et al. 2000), with the gravel cluster having the lowest porosity (average 27%; range 22–36%), sand cluster intermediate (average 40%; range 35–42%) and fines/diamicts cluster highest (average 44%; range 41–48%).

Hydraulic conductivity ranges for each cluster were estimated using the Kozeny–Carman equation. Gravel and sand clusters have higher *K* values (geometric means of 2.8 and 1.4 m/d, respectively) than fines/diamicts (geometric mean of 0.1 m/d). These results are consistent with previously measured values from pumping and slug tests. As the sand cluster *K* range is contained in the gravel cluster range, a potential bimodal hydrofacies definition may be appropriate when generating realizations of hydrofacies distributions for groundwater modelling, as has been suggested in other similar studies (e.g. Bianchi and Zheng 2016).

The methodology developed here is useful for classifying heterogeneous sediments for hydrogeological purposes when the particle size distribution can be measured, and facilitates consistent hydrogeological parameter assignment compared to a more traditional approach. Specifically, the clustering approach can be used to optimise hydrofacies definitions for the purposes of generating realisations for hydrogeological modelling using geostatistical methods. Site-specific conditions should be considered to adequately decide on the number of clusters and empirical relationships needed to infer hydraulic conductivity. Further studies should focus on (1) the comparison between different empirical equations and other methods by which *K* can be estimated, either in situ or laboratory based; and (2) the use of other easily measurable parameters, such as those obtained through geophysical measurements, to complement hydrofacies classifications and hydraulic conductivity estimates.

## Supplementary Information

Below is the link to the electronic supplementary material.

Supplementary file1 (PDF 485 KB)
