# Supplementary material for: Quantitative characterisation of Quaternary glaciofluvial aquifer heterogeneity using cluster analysis
Source: Hydrogeol J. 2025 Aug 2;33(5):1237–58. doi: 10.1007/s10040-025-02933-z (PMC12436504; doi:10.1007/s10040-025-02933-z)
Supplement: Supplementary file 1 — Supplementary file1 (PDF 485 KB) [file 10040_2025_2933_MOESM1_ESM.pdf]

Hydrogeology Journal: Electronic supplementary material

Quantitative characterisation of Quaternary glaciofluvial aquifer heterogeneity using cluster analysis

Felipe Gallardo Ceron[1,*], Landis Jared West[1,*], Ian T. Burke[2], James Graham[3] and Luca Colombera[4]

[1] Institute of Applied Geosciences, School of Earth and Environment, University of Leeds, Leeds, LS2 9JT, UK. * Corresponding Author's email: l.j.west@leeds.ac.uk (Landis Jared West), eefegc@leeds.ac.uk (Felipe Gallardo).

[2] Earth Surface Science Institute, School of Earth and Environment, University of Leeds, Leeds, LS2 9JT, UK.

[3] National Nuclear Laboratory Ltd., Sellafield, CA20 1PG, UK.

[4] Department of Earth and Environmental Sciences, University of Pavia, Italy

Electronic Supplementary Material

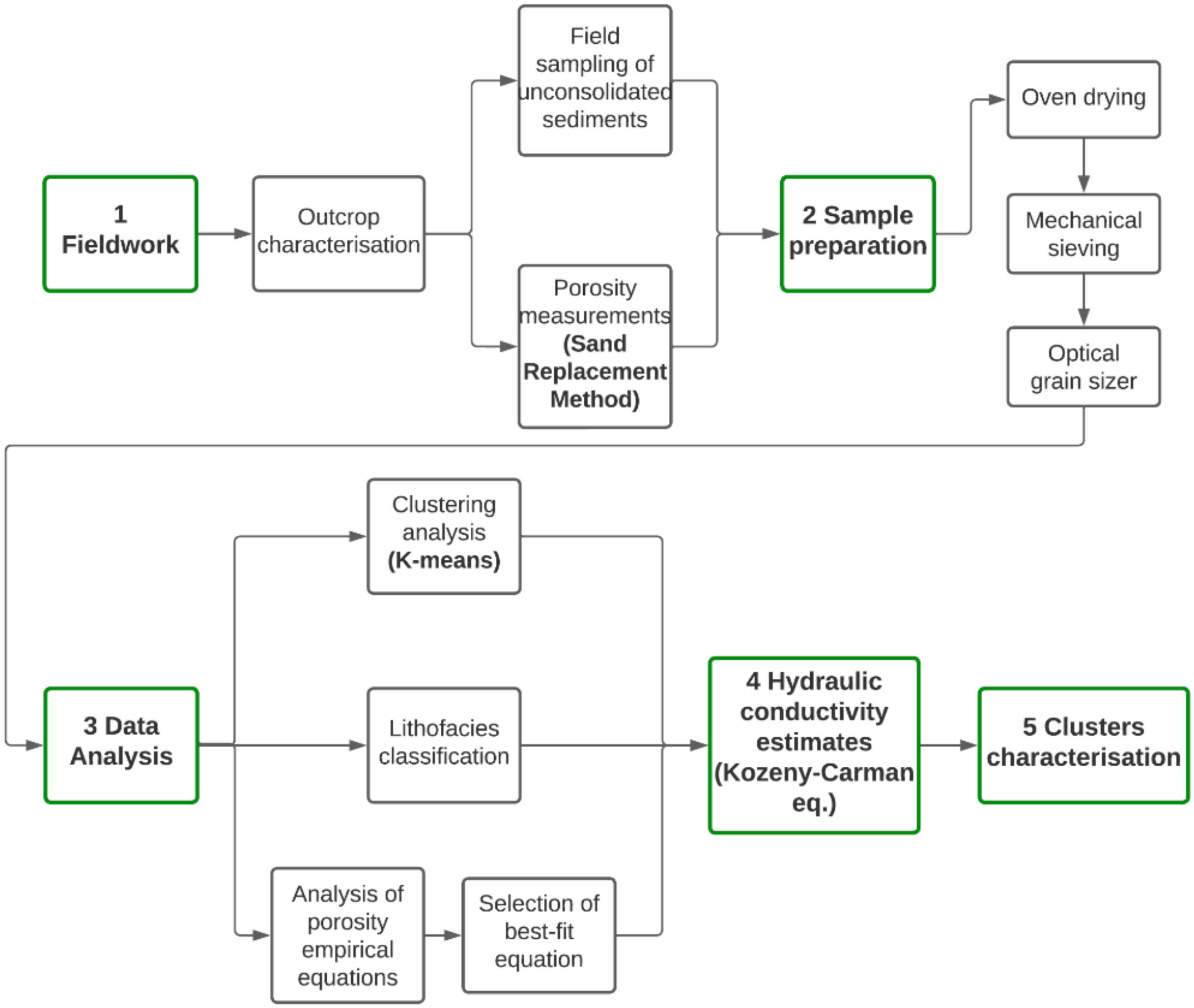

Figure S1. General workflow followed to characterise hydrofacies.

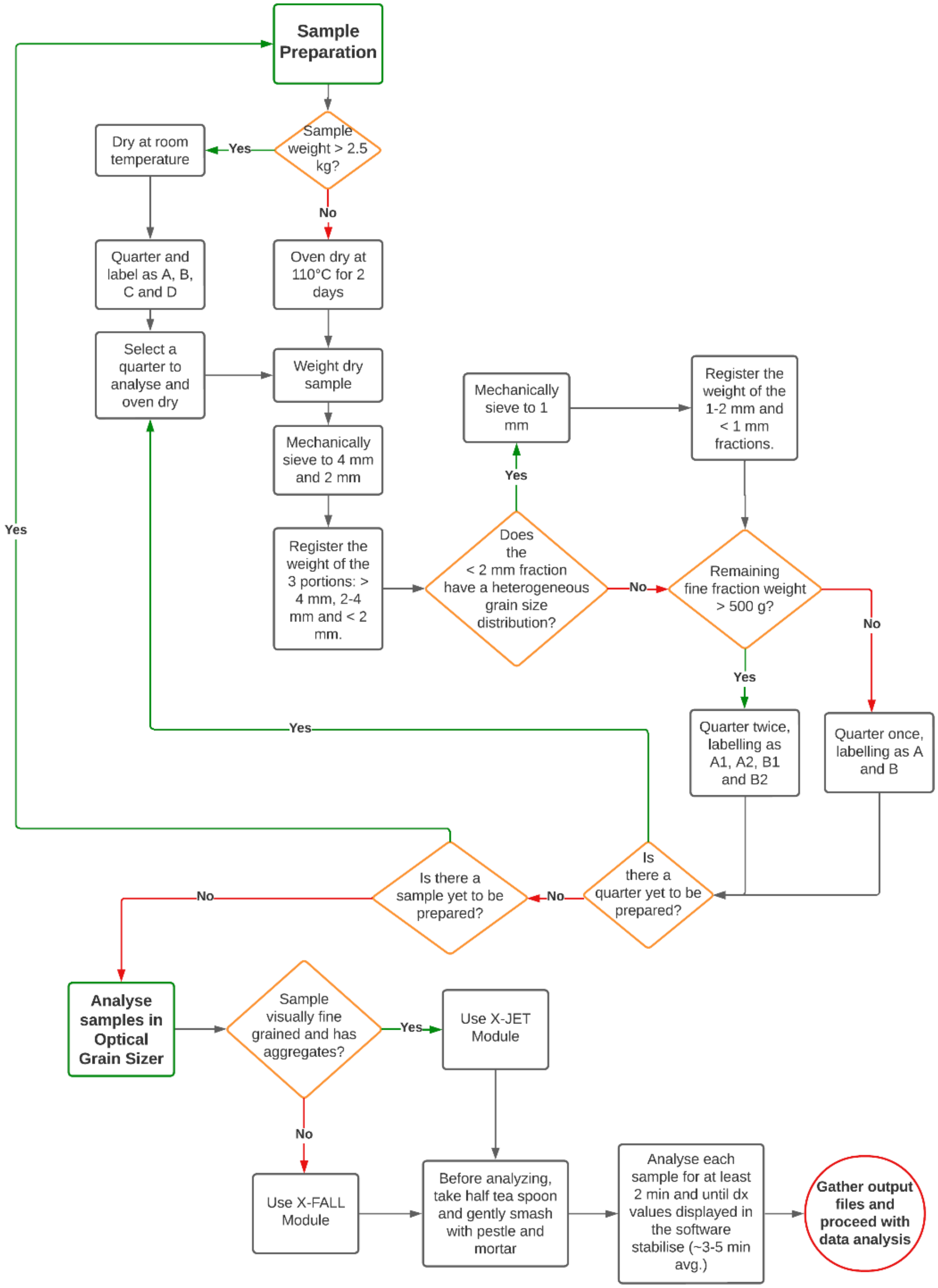

Figure S2. Detailed workflow followed to obtain the particle size distribution of unconsolidated sediments.

Table S1. Summary statistics of each cluster.

Variable	Measurement	Units	Fine-dominated cluster	Sand-dominated cluster	Gravel-dominated cluster
% Fine	Min-Max	%	43 - 93	6 - 42	2 - 22
	Avg		68	24	10
% Sand	Min-Max	%	7 - 53	54 - 94	8 - 50
	Avg		29	75	25
% Gravel	Min-Max	%	0 - 26	0 - 20	36 - 88
	Avg		3	2	65
d_{10} (mm)	Min-Max	mm	0.005 - 0.03	0.01 - 0.11	0.03 - 0.6
	Avg		0.010	0.04	0.16
	Median		0.008	0.03	0.08
d_{50} (mm)	Min-Max	mm	0.012 - 0.08	0.07 - 0.53	0.43 - 27
	Avg		0.040	0.16	9.3
	Median		0.036	0.13	6.9
d_{60} (mm)	Min-Max	mm	0.017 - 0.12	0.09 - 0.65	0.77 - 34
	Avg		0.055	0.19	13.8
	Median		0.049	0.15	10.4
U	Min-Max	-	2.1 - 16	2.7 - 12	15 - 1057
	Avg		6.3	5.6	175
	Median		4.7	4.8	95
	Geom Mean		5.4	5.2	105
Porosity	Min-Max	-	0.41 - 0.48	0.35 - 0.42	0.22 - 0.36
	Avg		0.441	0.394	0.270
	Median		0.440	0.398	0.263
	Geom Mean		0.440	0.394	0.267
K^*	Min-Max	m/d	6.4E-02 – 1.9E-01	1.8E-01 - 1.1E+01	1.2E-01 - 6.2E+01
	Avg		1.0.E-01	2.5.E+00	9.5.E+00
	Median		8.4.E-02	1.1.E+00	2.2.E+00
	Geom Mean		1.0.E-01	1.4.E+00	2.8.E+00

* Hydraulic conductivity values for fine dominated cluster excluding outliers

Table S2. List of field samples and their corresponding lithofacies, average percentage of fines, sand and gravel; cluster classification, average porosity and average hydraulic conductivity.

Sample Name	Lithofacies	Number of lab analyses	Average % of Fine	Average % of Sand	Average % of Gravel	Cluster	Avg. Porosity (d_{50} eq)	Avg. *K* (d_{50} porosity)
FG_01	Sp	4	8%	92%	0%	Cluster 2 - Sand	0.374	1.1.E+01
FG_02	Sh	4	6%	94%	0%	Cluster 2 - Sand	0.373	1.1.E+01
FG_03	Sh	4	26%	75%	0%	Cluster 2 - Sand	0.400	1.6.E+00
FG_04	Fm	2	58%	42%	0%	Cluster 1 - Fine	0.436	8.0.E-02
GB_01	-	2	74%	26%	0%	Cluster 1 - Fine	0.440	1.9.E-01
GB_02	-	2	82%	16%	2%	Cluster 1 - Fine	0.459	8.1.E-02
GB_03	-	10	10%	25%	66%	Cluster 3 - Gravel	0.279	1.9.E+00
GB_04	-	8	13%	21%	67%	Cluster 3 - Gravel	0.273	6.6.E-01
GB-SampleD	-	2	68%	32%	0%	Cluster 1 - Fine	0.440	1.9.E-01
GB-SampleE	-	2	47%	50%	3%	Cluster 1 - Fine	0.417	1.3.E-01
IB_02	-	4	13%	37%	51%	Cluster 3 - Gravel	-	-
IB_03	-	4	38%	63%	0%	Cluster 2 - Sand	0.413	1.4.E+00
IB_04	-	4	41%	60%	0%	Cluster 2 - Sand	0.413	1.0.E+00
IB_05	-	2	64%	36%	0%	Cluster 1 - Fine	0.439	1.5.E-01
PQ-01	Sh	2	27%	73%	0%	Cluster 2 - Sand	0.404	4.0.E-01
PQ-02	Gcm	2	4%	16%	80%	Cluster 3 - Gravel	0.236	2.3.E+01
PQ-03	Gcm	3	3%	34%	63%	Cluster 3 - Gravel	0.268	2.4.E+01
PQ-04	Sp	2	23%	72%	5%	Cluster 2 - Sand	0.379	1.1.E+00
PQ-05	Sm	2	33%	65%	2%	Cluster 2 - Sand	0.403	4.2.E-01
PQ-06	Gcm	2	4%	15%	81%	Cluster 3 - Gravel	0.263	3.1.E+01
PQ-07	Sp	2	9%	88%	3%	Cluster 2 - Sand	0.394	5.1.E+00
PQ-08	Sm	4	9%	90%	1%	Cluster 2 - Sand	0.369	8.5.E+00
PQ-09	Sm	2	41%	57%	2%	Cluster 2 - Sand	0.411	3.8.E-01
PQ-10	Gcm	2	5%	23%	73%	Cluster 3 - Gravel	0.240	1.3.E+01
PQ-11	Sh	4	35%	65%	0%	Cluster 2 - Sand	0.408	6.1.E-01
PQ-12	Gcm	4	13%	46%	41%	Cluster 3 - Gravel	0.343	1.8.E+00
PQ-13	Gcm	2	2%	17%	80%	Cluster 3 - Gravel	0.244	6.2.E+01
PQ-14	Sm	4	15%	85%	0%	Cluster 2 - Sand	0.392	3.2.E+00
Sample01	Fm	2	59%	42%	0%	Cluster 1 - Fine	0.424	-
Sample02	Fm	2	40%	60%	1%	Cluster 2 - Sand	0.415	1.8.E-01
Sample03	Sm	2	8%	73%	20%	Cluster 2 - Sand	0.350	6.8.E+00
Sample04	Gh	4	16%	26%	57%	Cluster 3 - Gravel	0.276	3.5.E-01
Sample05	Dmm	3	65%	32%	3%	Cluster 1 - Fine	0.447	8.3.E-02
Sample06	Sm	2	19%	82%	0%	Cluster 2 - Sand	0.395	1.7.E+00
Sample07	Fm	2	49%	51%	0%	Cluster 1 - Fine	0.421	-
Sample08	Sh	2	18%	81%	1%	Cluster 2 - Sand	0.385	1.1.E+00
Sample09	Fm	2	57%	41%	2%	Cluster 1 - Fine	0.426	-
Sample10	Fm	2	80%	15%	4%	Cluster 1 - Fine	0.470	6.4.E-02

Sample11	Fm	2	48%	51%	0%	Cluster 1 - Fine	0.418	6.6.E-02
Sample12	Sm	2	36%	64%	0%	Cluster 2 - Sand	0.414	6.0.E-01
Sample13	Fm	2	80%	20%	0%	Cluster 1 - Fine	0.460	8.5.E-02
Sample14	Gcm	2	22%	15%	62%	Cluster 3 - Gravel	0.255	2.2.E-01
Sample15	Fl	2	47%	53%	0%	Cluster 1 - Fine	0.418	-
Sample16	Fl	2	58%	41%	0%	Cluster 1 - Fine	0.424	-
Sample17	Gcm	4	11%	25%	64%	Cluster 3 - Gravel	0.271	2.9.E+00
Sample18	Fm	2	77%	23%	0%	Cluster 1 - Fine	0.455	9.0.E-02
Sample19	Fm	2	77%	23%	1%	Cluster 1 - Fine	0.457	7.8.E-02
Sample20	Sh	2	20%	80%	0%	Cluster 2 - Sand	0.395	1.1.E+00
Sample21	Sh	2	24%	77%	0%	Cluster 2 - Sand	0.402	1.8.E+00
Sample22	Sh	2	9%	92%	0%	Cluster 2 - Sand	0.386	5.1.E+00
Sample23	Sm	2	38%	62%	0%	Cluster 2 - Sand	0.411	9.8.E-01
Sample24	Sm	2	10%	90%	0%	Cluster 2 - Sand	0.378	3.8.E+00
Sample25	Dmm	2	64%	29%	7%	Cluster 1 - Fine	0.448	8.0.E-02
Sample26	Fm	2	90%	10%	0%	Cluster 1 - Fine	0.476	6.5.E-02
Sample27	Sm	2	26%	74%	0%	Cluster 2 - Sand	0.404	2.7.E-01
Sample28	Gp	4	7%	20%	73%	Cluster 3 - Gravel	0.263	3.7.E+00
Sample29	Gp	4	18%	20%	63%	Cluster 3 - Gravel	0.277	3.2.E-01
Sample30	Gcm	4	13%	32%	55%	Cluster 3 - Gravel	0.294	8.3.E-01
Sample31	Gh	4	20%	36%	44%	Cluster 3 - Gravel	0.326	4.0.E-01
Sample32	Gcm	4	5%	22%	73%	Cluster 3 - Gravel	0.257	1.6.E+01
Sample33	Gcm	-	-	-	-	-	-	-
Sample34	Fm	1	82%	18%	0%	Cluster 1 - Fine	0.433	1.8.E-01
Sample35	Fm	2	76%	24%	0%	Cluster 1 - Fine	0.429	-
Sample36	Fm	2	93%	7%	0%	Cluster 1 - Fine	0.456	8.1.E-02
Sample37	Fm	2	83%	17%	1%	Cluster 1 - Fine	0.461	8.1.E-02
Sample38	Gh	6	13%	39%	48%	Cluster 3 - Gravel	0.331	2.2.E+00
Sample39	Sm	2	18%	83%	0%	Cluster 2 - Sand	0.383	1.2.E+00
Sample40	Sm	2	23%	69%	9%	Cluster 2 - Sand	0.386	4.6.E-01
Sample41	Sm	2	32%	67%	1%	Cluster 2 - Sand	0.404	9.3.E-01
Sample42	Sm	2	22%	77%	1%	Cluster 2 - Sand	0.398	1.8.E+00
Sample43	Gh	4	14%	50%	36%	Cluster 3 - Gravel	0.357	2.1.E+00
Sample44	Gh	4	4%	17%	79%	Cluster 3 - Gravel	0.217	4.8.E+00
Sample45	Fl	2	76%	21%	2%	Cluster 1 - Fine	0.446	1.5.E-01
Sample46	Dmm	2	53%	28%	19%	Cluster 1 - Fine	0.426	1.0.E-01
Sample47	Gcm	-	-	-	-	-	-	-
Sample48	Fm	2	87%	13%	0%	Cluster 1 - Fine	0.450	1.1.E-01
Sample49	Sm	2	12%	88%	0%	Cluster 2 - Sand	0.363	2.9.E+00
Sample50	Gcm	2	6%	31%	64%	Cluster 3 - Gravel	0.219	2.6.E+00
Sample51	Fm	2	83%	16%	1%	Cluster 1 - Fine	0.469	6.7.E-02
Sample52	Gcm	-	-	-	-	-	-	-
Sample53	Gcm	3	5%	8%	87%	Cluster 3 - Gravel	0.226	2.0.E+01

Sample54	Gcm	2	4%	8%	88%	Cluster 3 - Gravel	0.244	2.1.E+01
Sample55	Fm	2	49%	48%	2%	Cluster 1 - Fine	0.417	7.9.E-02
Sample56	Fm	2	82%	18%	0%	Cluster 1 - Fine	0.450	1.7.E-01
Sample57	Gcm	4	15%	14%	71%	Cluster 3 - Gravel	0.225	1.2.E-01
Sample58	Sp	2	35%	64%	1%	Cluster 2 - Sand	0.405	6.2.E-01
Sample59	Gcm	4	14%	44%	42%	Cluster 3 - Gravel	0.331	1.1.E+00
Sample60	Sh	4	42%	54%	4%	Cluster 2 - Sand	0.415	9.8.E-01
Sample61	Gcm	3	43%	30%	26%	Cluster 1 - Fine	0.412	8.3.E-02
Sample62	Gcm	4	8%	16%	76%	Cluster 3 - Gravel	0.239	1.0.E+00
Sample63	Dmm	2	60%	33%	7%	Cluster 1 - Fine	0.436	1.2.E-01
Sample64	Fm	2	66%	34%	0%	Cluster 1 - Fine	0.440	1.1.E-01
Sample65	Gcm	-	-	-	-	-	-	-

Table S3. Summary of *K* values (m/d) reported by different authors.

Published in	Lithology	Facies	Cluster analysis equivalent	Minimum	Maximum	Arithmetic mean	Geometric mean	Measurement method	Other references
McMillan et al. (2000)	Silt/clays	Glaciolacustrine	Fines	1.7.E-05	3.5.E-02	4.4.E-03	1.2.E-03	Cone Penetrometer	Nirex 1997a
McMillan et al. (2000)	Sand	Glaciolacustrine	Sand	1.0.E-01	1.2.E+01	5.1.E+00	2.4.E+00	Cone Penetrometer	Nirex 1997a
McMillan et al. (2000)	Gravel	Glaciolacustrine	Gravel	3.0.E+00	3.2.E+01	2.0.E+01	1.3.E+01	Cone Penetrometer	Nirex 1997a
McMillan et al. (2000)	Basal sands	Glaciolacustrine	Sand	2.3.E-02	6.7.E+00	1.1.E+00	3.6.E-01	Slug test	Nirex 1997a
McMillan et al. (2000)	Multi-till sequence	Glaciolacustrine	General	5.2.E-03	1.0.E+02	5.1.E+00	4.3.E-01	Slug test	Nirex 1997a
McMillan et al. (2000)	Sands and gravels	Buried channel	Mixed (Sand, Gravel)	4.0.E-01	8.6.E+01	1.2.E+01	4.7.E+00	Pump test	Nirex 1997a
McMillan et al. (2000)	Silt	Buried channel	Fines	7.0.E-03	5.0.E-02	2.9.E-02	1.9.E-02	Pump test	Nirex 1997a
McMillan et al. (2000)	Gravel	Glaciofluvial	Gravel	3.2.E-02	2.6.E+01	3.8.E+00	5.0.E-01	Fair & Hatch	Nirex 1997a
McMillan et al. (2000)	Sand	Glaciofluvial	Sand	1.3.E-02	5.9.E-02	4.0.E-02	3.4.E-02	Fair & Hatch	Nirex 1997a
McMillan et al. (2000)	Sand	Glaciolacustrine	Sand	2.1.E-03	1.0.E-02	6.6.E-03	5.6.E-03	Fair & Hatch	Nirex 1997a
McMillan et al. (2000)	Silt	Glaciolacustrine	Fines	1.4.E-04	2.9.E-04	2.1.E-04	2.0.E-04	Fair & Hatch	Nirex 1997a
Smith et al. (2022)	Clay	n/a	Fines	5.0.E-07	1.0.E-03	-	-	General values	Lewis et al. (2006)
Smith et al. (2022)	Silt	n/a	Fines	1.0.E-03	1.0.E-01	-	-	General values	Lewis et al. (2006)
Smith et al. (2022)	Sand	n/a	Sand	1.0.E-01	1.0.E+02	-	-	General values	Lewis et al. (2006)
Smith et al. (2022)	Gravel	n/a	Gravel	5.0.E+01	5.0.E+04	-	-	General values	Lewis et al. (2006)
Smith et al. (2022)	Sand and gravel	n/a	Mixed (Sand, Gravel)	5.0.E+00	1.0.E+02	-	-	General values	Lewis et al. (2006)
Smith et al. (2022)	Till	n/a	Fines	1.0.E-07	5.0.E-01	-	-	General values	Lewis et al. (2006)
Purkis et al. (2023)	Clay	Mud	Fines	7.6.E-05	7.6.E-05	7.6.E-05	-	Consolidation method (lab)	
Purkis et al. (2023)	Sand	Gravelly sand	Sand	1.3.E+02	1.3.E+02	1.3.E+02	-	Falling-head (lab)	
Purkis et al. (2023)	Gravel	Sandy gravel (loose)	Gravel	3.9.E+02	3.9.E+02	3.9.E+02	-	Falling-head (lab)	
Purkis et al. (2023)	Gravel	Sandy gravel (dense)	Gravel	5.9.E+02	5.9.E+02	5.9.E+02	-	Falling-head (lab)	
Nirex (1997b)	Whole aquifer	n/a	General	2.1.E-01	9.5.E+01	8.4.E+00	1.9.E+00	Radial flow modelling	
McDonald et al (2012)	Silt	Glaciofluvial	Fines	6.0.E-03	4.2.E-02	2.4.E-02	1.6.E-02	Guelph permeameter	
McDonald et al (2012)	Sand	Glaciofluvial and glaciolacustrine	Sand	4.8.E-02	3.0.E+01	6.7.E+00	1.8.E+00	Guelph permeameter	
McDonald et al (2012)	Gravel	Glaciolacustrine and till	Gravel	1.8.E+00	3.1.E+01	1.3.E+01	6.7.E+00	Guelph permeameter	
This study	Silts	Glaciofluvial	Fines	6.4.E-02	1.9.E-01	1.0.E-01	1.0.E-01	Kozeny-Carman	
This study	Sands	Glaciofluvial	Sands	1.8E-01	1.1E+01	2.5.E+00	1.4.E+00	Kozeny-Carman	
This study	Gravels	Glaciofluvial	Gravel	1.2E-01	6.2E+01	9.5.E+00	2.8.E+00	Kozeny-Carman	
